# Association between hydrocortisone use and outcomes in sepsis patients with hospital-acquired bloodstream infections: An ancillary analysis of the EUROBACT-2 cohort

**DOI:** 10.1371/journal.pone.0356721

**Published:** 2026-09-15

**Authors:** Gary Duclos, Mohamed Boucekine, Alexis Tabah, Stéphane Ruckly, Niccolò Buetti, Jean François Timsit, Marc Leone, EUROBACT-2 Study Group, ESICM, ESCMID ESGCIP, the OUTCOMEREA Network

**Affiliations:** 1 Department of Anesthesiology and Intensive Care Unit, Hospital Nord, Assistance Publique Hôpitaux Universitaires de Marseille, Marseille, France; 2 Centre d’Etudes et de Recherches sur les services de santé et qualité de vie EA 3279, Aix Marseille Université, Marseille, France; 3 Intensive Care Unit, Redcliffe Hospital, Brisbane, Australia; 4 Queensland University of Technology, Brisbane, Queensland, Australia; 5 Faculty of Medicine, The University of Queensland, Brisbane, Queensland, Australia; 6 Université de Paris, INSERM, IAME UMR 1137, Paris, France; 7 ICUREsearch, Biometry, Fontaine, France; 8 Infection Antimicrobials Modeling Evolution U 1137, INSERM, Université Paris-Cité, Paris, France; 9 Infection Control Program and WHO Collaborating Centre on Patient Safety, Geneva University Hospitals and Faculty of Medicine, Geneva, Switzerland; 10 Medical and Infectious Diseases Intensive Care Unit, AP-HP, Bichat-Claude Bernard University Hospital, Paris, France; 11 OUTCOME REA network, Drancy, France; 12 Aix Marseille University, Marseille, France; Nitte University, INDIA

## Abstract

**Purpose:**

Corticosteroids are suggested for selected patients with septic shock, but their association with outcomes has not been specifically investigated in patients with hospital-acquired bloodstream infections (HA-BSI). We explored the association between hydrocortisone use and day-28 mortality in this population.

**Methods:**

Data from the prospective EUROBACT-2 study were used to assess the association between hydrocortisone use and day-28 mortality in ICU patients with HA-BSI and sepsis. Patients receiving hydrocortisone were compared with patients not receiving hydrocortisone. A 1:1 propensity score matching procedure was performed using nearest-neighbour matching without replacement and a caliper of 0.2 standard deviation of the logit propensity score, followed by multivariable logistic regression with cluster-robust standard errors by matched pair.

**Results:**

Among 2,401 analyzed patients, 608 (25%) received hydrocortisone during the HA-BSI episode. The final propensity score included age, sex, Charlson comorbidity score, ICU admission diagnosis, SOFA score without cardiovascular component, septic shock, country of inclusion, lactate at HA-BSI diagnosis, and ICU-acquired versus non-ICU-acquired HA-BSI. Matching yielded 521 pairs (1,042 patients). In the multivariable model, hydrocortisone use was associated with increased day-28 mortality in the overall matched cohort (OR 1.448; 95% CI 1.096–1.914; p = 0.009). This association was observed in patients with sepsis without shock (OR 1.697; 95% CI 1.051–2.740; p = 0.031) and in the female subgroup (OR 1.718; 95% CI 1.055–2.796; p = 0.030), but not in patients with septic shock or in those receiving norepinephrine-equivalent dose ≥0.25 µg/kg/min.

**Conclusion:**

Hydrocortisone use was not associated with improved day-28 survival in ICU patients with HA-BSI. In the matched analysis, hydrocortisone use was associated with higher day-28 mortality in the overall cohort, whereas no significant association was observed in patients with septic shock or high-dose norepinephrine. These findings are observational and hypothesis-generating and should not be interpreted causally.

## Introduction

The Surviving Sepsis Campaign (SSC) guidelines suggest intravenous hydrocortisone, usually 200 mg daily, for adults with septic shock and an ongoing vasopressor requirement. The 2021 SSC guidelines refer to a norepinephrine or epinephrine dose ≥0.25 µg/kg/min for at least 4 hours after initiation as a clinically relevant threshold for vasopressor requirement [1]. Although corticosteroids shorten shock duration in septic shock, their effect on mortality remains uncertain. Furthermore, major randomized trials of corticosteroids in septic shock or severe infections included heterogeneous populations, and microbiological documentation was incomplete in a substantial proportion of patients [2–6]

The EUROBACT-2 international cohort prospectively enrolled 2,600 adult patients with HA-BSI managed in 333 ICUs across 52 countries. Because inclusion required a positive blood culture, all patients had microbiologically documented infection [7]. HA-BSI represents a late-onset, hospital-acquired and microbiologically documented infectious context that differs from many randomized corticosteroid trials. We used this database to explore the association between hydrocortisone use and outcomes in critically ill patients with HA-BSI, while accounting for measured severity, centre/country effects, and potential confounding by indication.

## Methods

From the EUROBACT-2 database, we included adult ICU patients with HA-BSI, defined as a positive blood culture sample taken more than 48 hours after hospital admission. HA-BSI had to be managed in the ICU, either because it was acquired in the ICU or because the patient was transferred to the ICU for management of the infection [7].

All eligible patients with HA-BSI from the EUROBACT-2 cohort were considered. Hydrocortisone administration during the HA-BSI episode defined the exposed group, whereas patients not receiving hydrocortisone served as controls. Patients with chronic corticosteroid use of more than 20 mg per day for at least four weeks and patients receiving a corticosteroid other than hydrocortisone during the ICU stay were excluded according to the EUROBACT-2 study definitions.

### Ethics statement

Initial ethical approval as a low-risk research project with waiver of individual consent was granted by the Human Research Ethics Committee of the Royal Brisbane & Women’s Hospital, Queensland, Australia (LNR/2019/QRBW/48376). Each study site then obtained ethical and governance approvals according to national and/or local regulations.

### Primary goal

Our primary objective was to assess the association between hydrocortisone use during the HA-BSI episode and day-28 mortality in ICU patients with HA-BSI and sepsis.

### Secondary goals

Secondary objectives were to assess the association between hydrocortisone use and day-28 mortality in predefined subgroups: patients with HA-BSI-related septic shock, patients with HA-BSI-related sepsis without shock, female patients, and patients receiving norepinephrine-equivalent dose ≥0.25 µg/kg/min at HA-BSI diagnosis. Exploratory secondary outcomes included vasopressor-free days at day 28, mechanical ventilation-free days at day 28, and SOFA variation between HA-BSI diagnosis and day 7.

### Patient characteristics

- Demographic data, main diagnosis at admission to the ICU, and comorbidities were collected. The Charlson score was used as a continuous variable.
- Illness severity was assessed using the Simplified Acute Physiology Score II (SAPS II) and the Sequential Organ Failure Assessment (SOFA) score. The SOFA score without the cardiovascular component was calculated. Sepsis and septic shock were defined according to Sepsis-3 criteria on the day of blood culture sampling [1].
- The maximum dose of norepinephrine equivalent was calculated in µg/kg/min. For patients who acquired HA-BSI in the ICU, the highest dose administered on the day of HA-BSI diagnosis, which was defined as the day of blood culture sampling, was recorded. For those who acquired HA-BSI prior to ICU admission, the maximum dose administered on the first day of ICU stay was recorded.
- The maximum dose of norepinephrine equivalent was calculated by adding a dose of norepinephrine and epinephrine using a 1:1 dose equivalence [8,9].
- A norepinephrine-equivalent dose threshold of ≥0.25 µg/kg/min was used to define high-dose norepinephrine requirement. This threshold was considered a clinically relevant vasopressor-dose threshold in line with SSC guidance, rather than a formal definition of septic shock [1].

### Characteristics of HA-BSI

- The sources of HA-BSI were categorized into six groups (catheter-related, respiratory, intra-abdominal, urinary, primary HA-BSI, and others) determined by the senior physician in charge. For patients with more than one possible source of HA-BSI, the sources were classified according to the most probable locus of infection.
- The time for adequate administration of empirical therapy was defined as the time in hours between HA-BSI and the administration of at least one antibiotic agent effective on the responsible pathogen, according to its antibiogram and infection source. The time of reference was when blood culture sampling was undertaken.
- Source control was defined as the performance of the measures required to treat the origin of the HA-BSI (*e.g.,* percutaneous, surgical drainage, and catheter removal). Source control was classified into three categories according to the treating clinician: *1)* source control measures were not indicated, *2)* source control measures were indicated and appropriately performed, and *3)* source control measures were indicated but were neither performed nor partially performed.
- HA-BSI was considered acquired after ICU admission when the first positive blood culture was obtained more than 48 hours after ICU admission.

### Characteristics of hydrocortisone use

For each patient, hydrocortisone administration and number of treatment days were recorded. However, the exact date and time of hydrocortisone initiation relative to blood culture sampling or HA-BSI diagnosis were not available. Each centre reported its usual protocol regarding hydrocortisone indications, dosage, and association with fludrocortisone, but details on the exact daily dose were not reported at the patient level.

### Centre characteristics

Country and income categories were determined according to United Nations M49 standards. Clinical pharmacists’ consultations were categorized as follows in the original study [7]: “available during business hours,” “available on request 24/7,” “available as part of permanent ICU staff,” “available on scheduled meeting,” and “never consulted.” For this study, this outcome was assessed as “any consultation with a clinical pharmacist,” as opposed to no consultation with a clinical pharmacist.

### Statistical analysis

Baseline characteristics are presented for the full enrolled sample and for each subgroup. The Wilcoxon rank-sum test and the chi-square test were used to test for differences for continuous and categorical variables, respectively. A Fisher’s exact test was used for categorical variables with individual cell counts below five.

For all calculations, the time of reference was the moment of the first positive blood culture sampling. Vital status on day 28 after the first positive blood culture sampling was collected. Exposure to invasive therapies (mechanical ventilation, vasopressors, renal replacement therapy, and extra-corporeal membrane oxygenation therapy) was also noted. To prevent exposure bias, exposure-free days were calculated for survivors on day 28. All patients who died during the period had an exposure-free day from invasive therapies, calculated as equal to zero.

Directed acyclic graphs (DAGs) were used to identify significant variables to include in propensity score matching and the final multivariate model. DAGs, or causal diagrams, are graphical representations of causal structures that help to understand and illustrate potential biases, including biases arising from confounding, selection, and misclassification [10] (S1 Fig).

A propensity score model estimating the probability of receiving hydrocortisone was developed using logistic regression and variables selected from the DAGs and clinically relevant baseline characteristics [11]. Matching was performed using 1:1 nearest-neighbour matching without replacement and a caliper width of 0.20 standard deviation of the logit of the propensity score [11,12]. Patients outside the region of common support were not included in the matched analysis.

The final propensity score included age, sex, Charlson comorbidity score, diagnosis at ICU admission, SOFA score without cardiovascular component at HA-BSI diagnosis, septic shock, country of inclusion, lactate at HA-BSI diagnosis, and ICU-acquired versus non-ICU-acquired HA-BSI. Matching quality was assessed using propensity score distribution plots and standardized mean differences, with SMD < 0.1 considered indicative of good balance.

The primary multivariable logistic regression model assessed the association between hydrocortisone exposure and day-28 mortality in the matched sample. Covariates included respiratory source of infection, time to adequate antimicrobial therapy, source control category, clinical pharmacist never consulted, centre use of fludrocortisone, and norepinephrine-equivalent dose ≥0.25 µg/kg/min at HA-BSI diagnosis. Hydrocortisone duration was removed from the primary model because it occurred after treatment initiation and could be influenced by subsequent clinical evolution and survival.

Predefined subgroup analyses were performed in patients with septic shock at HA-BSI diagnosis, patients with sepsis without shock, female patients, and patients receiving norepinephrine-equivalent dose ≥0.25 µg/kg/min at HA-BSI diagnosis. The formal hydrocortisone × sex interaction was also assessed to avoid overinterpretation of sex-specific subgroup findings.

### Sensitivity analysis

Sensitivity analyses were conducted to assess the robustness of the findings. First, patients admitted to the ICU for COVID-19 were excluded. Second, time to adequate antimicrobial therapy and source control were removed from the multivariable model because these variables may occur after baseline and may be influenced by clinical evolution. Third, patients with coagulase-negative staphylococcal bloodstream infection were excluded from the already matched cohort without rematching.

Exploratory secondary outcomes were analyzed according to hydrocortisone exposure. Vasopressor-free days and mechanical ventilation-free days at day 28 were analyzed, as was the change in SOFA score between HA-BSI diagnosis and day 7. For delta SOFA, negative values indicated improvement and positive values indicated worsening between HA-BSI diagnosis and day 7.

Multivariable analyses were performed in the matched cohort and predefined subgroups using the same adjustment strategy. Logistic regression models were used for day-28 mortality and linear regression models for exploratory continuous secondary outcomes. Cluster-robust standard errors by matched pair were used. Results were expressed as odds ratios (ORs) with 95% confidence intervals (CIs) for mortality and as adjusted mean differences (β) with 95% CIs for secondary outcomes. Statistical analyses were performed using R (R Core Team (2020), R: A language and environment for statistical computing) and Python for additional figure and table generation.

## Results

### Patient characteristics

**Fig 1:**
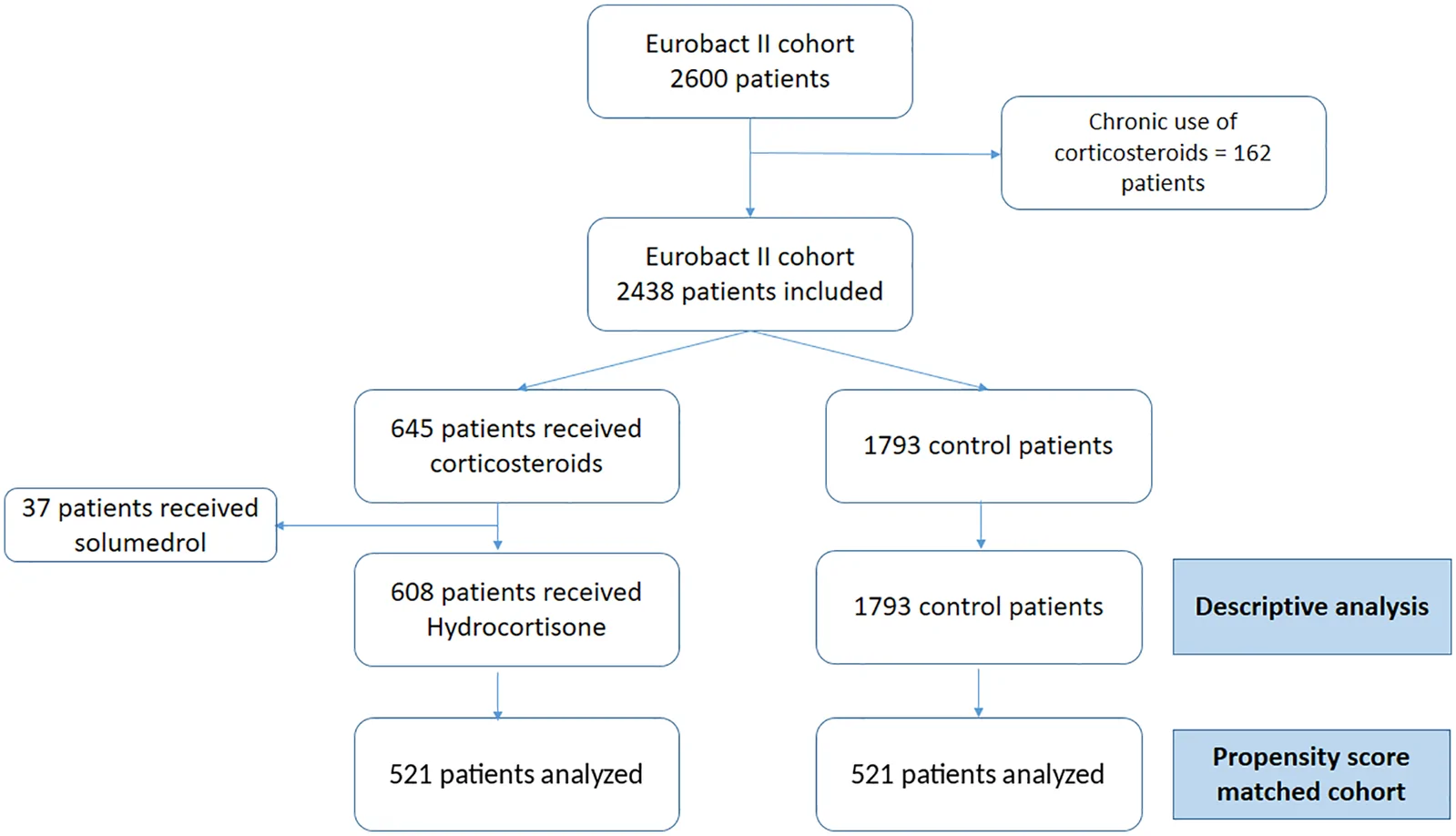
Flowchart.

The EUROBACT-2 cohort included 2,600 patients, of whom 162 were excluded because they received chronic corticosteroid treatment (Fig 1). Among the 2,438 remaining patients, 37 were excluded because they received corticosteroids other than hydrocortisone during the ICU stay. Among the 2,401 analyzed patients, 608 (25%) received hydrocortisone during the HA-BSI episode (Fig 1). Patient characteristics are reported in Table 1, with additional details in S1–S4 Tables. The mean age was 61 ± 16 years, and 1,543 patients (64%) were male. At ICU admission, the mean SAPS II and Charlson score were 49 ± 17 and 2.27 ± 2.57, respectively. The mean interval between hospital admission and ICU admission was 8.5 ± 22.6 days, and the mean time between hospital admission and HA-BSI diagnosis was 18 ± 29 days. HA-BSI was diagnosed before ICU admission in 500 (21%) patients, during the first 48 hours of ICU admission in 210 (8.8%) patients, and more than 48 hours after ICU admission in 1,685 (70%) patients.

**Table 1:** Main characteristics of the cohort. Values are n (%) or mean (SD). The corrected matched cohort corresponds to the final propensity score matching used in the analyses: 521 pairs, 1,042 patients.

	Before matching				After matching			
Characteristic	Overall 2401 (100%)	Controls 1793 (74.6%)	Hydrocortisone 608 (25.3%)	p value	Overall 1042 (100%)	Controls 521 (50.0%)	Hydrocortisone 521 (50.0%)	p value
**Characteristics**								
Male gender	1,543 (64%)	1,161 (65%)	382 (63%)	0.4	674 (65%)	334 (64%)	340 (65%)	0.7
Age (years)	61 (16)	61 (16)	62 (16)	0.9	63 (16)	63 (16)	62 (16)	0.8
Charlson comorbidity score	2.27 (2.57)	2.20 (2.54)	2.48 (2.67)	0.007	2.50 (2.69)	2.52 (2.68)	2.48 (2.71)	0.8
**Patient status at ICU admission**								
Delay between hospital admission and ICU admission (days)	8.5 (23)	9.1 (30)	8.0 (17)	0.015	9 (22)	9.0 (17)	8.0 (17)	0.3
SAPS II score at ICU admission	49 (17)	48 (16)	53 (18)	<0.001	52 (18)	51 (17)	53 (18)	0.3
**Primary ICU admission diagnosis**								
Respiratory failure	495 (21%)	359 (20%)	136 (22%)	< 0.001	220 (21%)	112 (21%)	108 (21%)	0.4
Sepsis or septic shock	480 (20%)	312 (17%)	168 (28%)		287 (28%)	137 (26%)	150 (29%)	
Pandemic infection (confirmed or suspected)	314 (13%)	223 (12%)	91 (15%)		159 (15%)	80 (15%)	79 (15%)	
Neurologic causes	252 (10%)	223 (12%)	29 (4.8%)		54 (5.2%)	30 (5.8%)	24 (4.6%)	
Post-operative admission	244 (10%)	196 (11%)	48 (7.9%)		93 (8.9%)	49 (9.4%)	44 (8.4%)	
Cardiovascular causes	130 (5.4%)	99 (5.5%)	31 (5.1%)		47 (4.5%)	22 (4.2%)	25 (4.8%)	
Cardiac arrest	89 (3.7%)	69 (3.8%)	20 (3.3%)		32 (3.1%)	15 (2.9%)	17 (3.3%)	
Traumatic brain injury	93 (3.9%)	75 (4.2%)	18 (3.0%)		29 (2.8%)	13 (2.5%)	16 (3.1%)	
Multiple trauma (no TBI)	91 (3.8%)	68 (3.8%)	23 (3.8%)		42 (4.0%)	22 (4.2%)	20 (3.8%)	
Gastrointestinal causes	79 (3.3%)	65 (3.6%)	14 (2.3%)		29 (2.8%)	16 (3.1%)	13 (2.5%)	
Hypovolemic or hemorrhagic shock	44 (1.8%)	29 (1.6%)	15 (2.5%)		28 (2.7%)	15 (2.9%)	13 (2.5%)	
Renal failure	43 (1.8%)	38 (2.1%)	5 (0.8%)		11 (1.1%)	6 (1.2%)	5 (1.0%)	
Metabolic causes	43 (1.8%)	35 (2.0%)	8 (1.3%)		9 (0.9%)	3 (0.6%)	6 (1.2%)	
Other	4 (0.2%)	2 (0.1%)	2 (0.3%)		2 (0.2%)	1 (0.2%)	1 (0.2%)	
**Bloodstream infection characteristics**								
**Most likely source of infection**								
Respiratory	627 (26%)	439 (24%)	188 (31%)	0.013	278 (27%)	126 (24%)	152 (29%)	0.3
Catheter-related	649 (27%)	511 (28%)	138 (23%)		246 (24%)	129 (25%)	117 (22%)	
Primary	390 (16%)	301 (17%)	89 (15%)		178 (17%)	98 (19%)	80 (15%)	
Intra-abdominal	364 (15%)	263 (15%)	101 (17%)		181 (17%)	92 (18%)	89 (17%)	
Urinary	173 (7.2%)	127 (7.1%)	46 (7.6%)		71 (6.8%)	31 (6.0%)	40 (7.7%)	
Other	198 (8.2%)	152 (8.5%)	46 (7.6%)		88 (8.4%)	45 (8.6%)	43 (8.3%)	
**Control of the source of infection**								
Not required	1,131 (47%)	852 (48%)	279 (46%)	0.026	507 (49%)	264 (51%)	243 (47%)	0.8
Required and done	1,044 (43%)	789 (44%)	255 (42%)		421 (40%)	204 (39%)	217 (42%)	
Required but not done	226 (9.4%)	152 (8.5%)	74 (12%)		114 (11%)	53 (10%)	61 (12%)	
**Time to adequate antimicrobial therapy (hours)**								
Never	374 (17%)	268 (16%)	106 (18%)	0.049	180 (18%)	93 (19%)	87 (17%)	0.4
0–24 h	1,133 (51%)	817 (50%)	316 (54%)		535 (54%)	252 (52%)	283 (56%)	
24–48 h	276 (12%)	214 (13%)	62 (11%)		106 (11%)	49 (10%)	57 (11%)	
48–120 h	340 (15%)	265 (16%)	75 (13%)		126 (13%)	65 (14%)	61 (12%)	
≥120 h	104 (4.7%)	83 (5.0%)	21 (3.6%)		36 (3.7%)	22 (4.6%)	14 (2.8%)	
Associated septic shock at time of BSI	758 (32%)	421 (23%)	337 (55%)	<0.001	598 (57%)	298 (57%)	300 (58%)	0.8
Use of norepinephrine	1,230 (51%)	778 (43%)	452 (75%)	<0.001	761 (73%)	362 (70%)	399 (77%)	0.002
Norepinephrine dose equivalent (µg/kg/min)	0.23 (0.82)	0.17 (0.73)	0.44 (1.05)	<0.001	0.39 (1.01)	0.31 (0.93)	0.47 (1.08)	0.007
High dose of norepinephrine equivalent	328 (19%)	191 (14%)	137 (37%)	<0.001	219 (34%)	100 (30%)	119 (38%)	0.086
**Outcome at day 28**								
Vasopressor-free days	15 (12)	16 (12)	10 (11)	<0.001	11 (12)	13 (12)	10 (11)	0.08
Mechanical ventilation-free days	11 (12)	12 (12)	8 (11)	<0.001	9 (11)	10 (12)	8 (11)	0.01
Renal replacement therapy-free days	16 (13)	18 (13)	12 (13)	<0.001	13 (13)	14 (13)	12 (13)	0.2
ECMO-free days	18 (13)	19 (13)	14 (14)	<0.001	15 (14)	16 (14)	14 (14)	0.3
ICU-free days	7 (14)	8 (15)	4 (8)	0.070	6 (18)	8 (24)	4 (8)	0.2
Hospital-free days	2.0 (5.2)	2.3 (5.5)	1.1 (3.9)	<0.001	1.5 (4.6)	2.0 (5.3)	1.0 (3.6)	0.4
Dead at day 28	875 (36%)	570 (32%)	305 (50%)	<0.001	480 (46%)	220 (42%)	260 (50%)	<0.001
Dead in ICU at day 28	810 (34%)	519 (29%)	291 (48%)	<0.001	446 (43%)	199 (38%)	247 (47%)	<0.001

ICU = intensive care unit; BSI = bloodstream infection; SAPS = Simplified Acute Physiology Score; TBI = traumatic brain injury; ECMO = extracorporeal membrane oxygenation.

### Characteristics of corticosteroid use according to centre

Centre-level hydrocortisone practices are described in S5 Table. In 26 (8%) of 330 centres, hydrocortisone was never prescribed. In the other centres, usual indications for hydrocortisone included sepsis, septic shock, and septic shock with high-dose vasopressors. Among the 608 patients receiving hydrocortisone, most received a centre-reported regimen corresponding to 200 mg/day by intermittent bolus or continuous infusion. Hydrocortisone was usually administered until shock resolution or for a fixed 7-day course, according to centre practice.

### Outcome

Propensity score matching using the final propensity score model yielded 521 pairs, corresponding to 1,042 matched patients (Fig 1). The effect of matching on variables included in the propensity score, variables included in the multivariable model, centre/country/income variables, and additional clinically relevant variables is shown in S2–S4 Figs. A descriptive comparison of matched and unmatched patients is provided in S7 Table, supporting the interpretation that the matched analysis applies to the region of common support.

**Fig 2:**
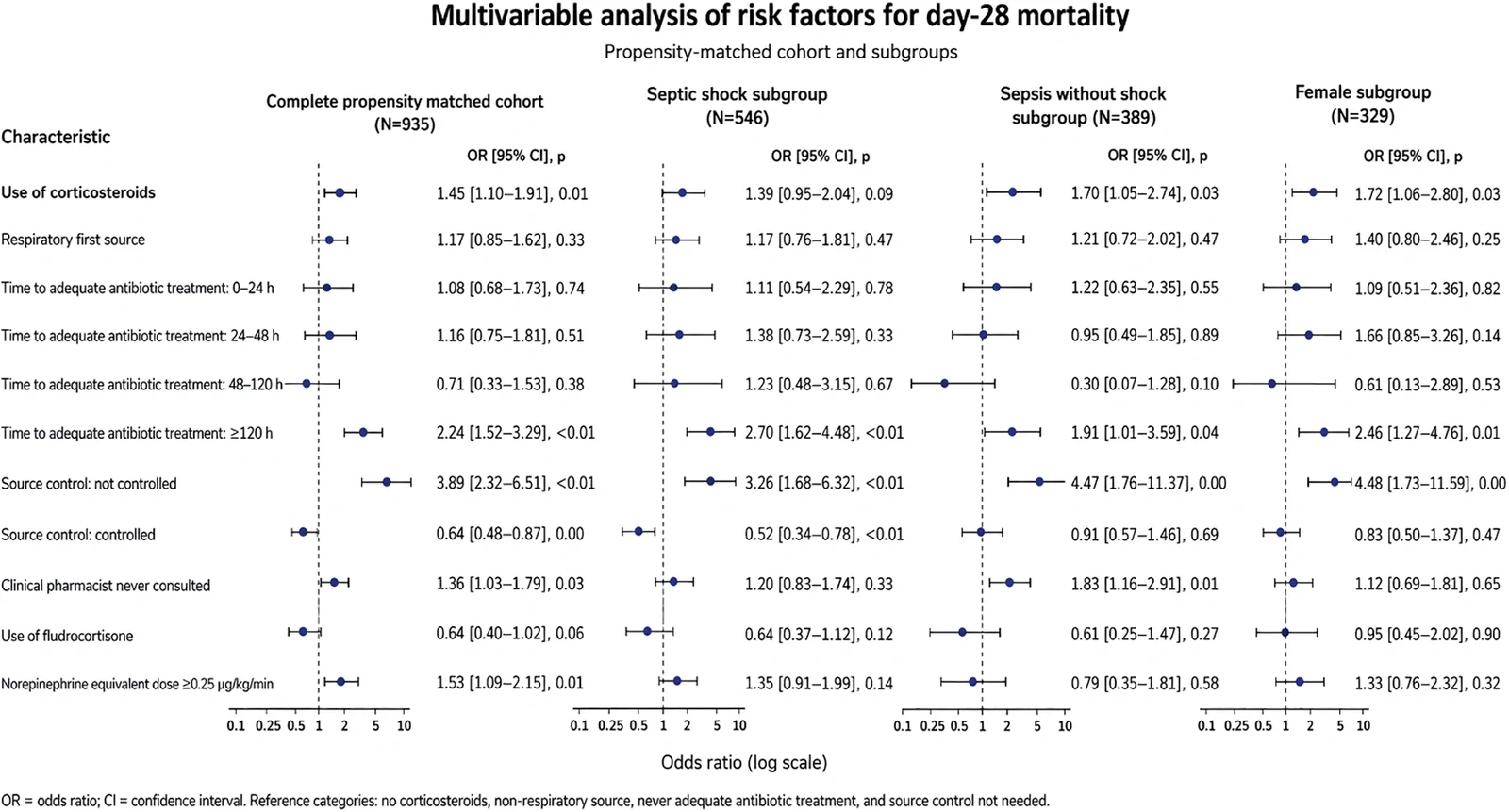
Forest plot of the multivariable analysis in the propensity-matched cohort and predefined subgroups.

Day-28 mortality was 36% (n = 875) in the unmatched cohort. In the matched cohort, day-28 mortality was 50% (260/521) in the hydrocortisone group and 42% (220/521) in the control group. In the multivariable analysis (N = 935), hydrocortisone use was associated with day-28 mortality in the overall matched cohort (OR 1.448; 95% CI 1.096–1.914; p = 0.009) (Fig 2 and Table 2).

**Table 2:** Multivariable analysis of risk factors for day-28 mortality in the propensity-matched cohort and subgroups. Propensity score included age, sex, Charlson comorbidity score, diagnosis at ICU admission, SOFA score without cardiovascular component, septic shock, country of inclusion, lactate at BSI diagnosis, and ICU-acquired versus non-ICU-acquired BSI. Matching used 1:1 nearest-neighbour matching without replacement with a caliper of 0.2 SD of the logit propensity score. Missing values were excluded from the analysis.

Characteristic	Complete propensity-matched cohort (N = 935)			Septic shock subgroup (N = 546)			Sepsis without shock subgroup (N = 389)			Female subgroup (N = 329)		
	OR	95% CI	p	OR	95% CI	p	OR	95% CI	p	OR	95% CI	p
Hydrocortisone exposure	1.448	1.096; 1.914	0.009	1.392	0.952; 2.036	0.088	1.697	1.051; 2.740	0.031	1.718	1.055; 2.796	0.030
Respiratory first source	1.173	0.849; 1.620	0.334	1.174	0.760; 1.813	0.469	1.209	0.723; 2.023	0.469	1.400	0.798; 2.455	0.240
**Time to adequate antimicrobial therapy**												
Never (reference)	—	—	—	—	—	—	—	—	—	—	—	—
0–24 h	1.082	0.677; 1.731	0.741	1.108	0.535; 2.292	0.783	1.220	0.634; 2.350	0.551	1.094	0.506; 2.364	0.819
24–48 h	1.161	0.745; 1.807	0.510	1.377	0.733; 2.585	0.320	0.952	0.489; 1.853	0.885	1.660	0.846; 3.256	0.140
48–120 h	0.714	0.334; 1.525	0.384	1.229	0.479; 3.150	0.668	0.302	0.072; 1.279	0.104	0.607	0.127; 2.898	0.532
≥120 h	2.235	1.516; 3.294	<0.001	2.697	1.622; 4.484	<0.001	1.907	1.014; 3.586	0.045	2.459	1.272; 4.755	0.007
**Source control**												
Not needed (reference)	—	—	—	—	—	—	—	—	—	—	—	—
Not controlled	3.887	2.322; 6.509	<0.001	3.258	1.679; 6.324	<0.001	4.473	1.760; 11.365	0.002	4.475	1.727; 11.595	0.002
Controlled	0.645	0.478; 0.871	0.004	0.515	0.339; 0.784	0.002	0.908	0.566; 1.457	0.689	0.831	0.501; 1.377	0.472
Clinical pharmacist never consulted	1.357	1.027; 1.793	0.032	1.203	0.831; 1.741	0.328	1.835	1.158; 2.907	0.010	1.119	0.690; 1.813	0.648
Centre use of fludrocortisone	0.640	0.403; 1.015	0.058	0.643	0.371; 1.116	0.117	0.610	0.253; 1.469	0.270	0.954	0.450; 2.022	0.902
Norepinephrine equivalent dose ≥0.25 µg/kg/min	1.533	1.092; 2.152	0.014	1.347	0.910; 1.994	0.137	0.789	0.345; 1.808	0.576	1.329	0.761; 2.320	0.317

OR = odds ratio; CI = confidence interval; BSI = bloodstream infection. For time to adequate antimicrobial therapy, the reference category is ‘never’. For source control, the reference category is ‘not needed’. Estimates were obtained from logistic regression models with cluster-robust standard errors by matched pair. Subgroup models omit covariates that are constant within the subgroup. N refers to patients included in the complete-case multivariable model.

### Subgroup analyses

Among the matched patients, 598 had septic shock at HA-BSI diagnosis. Day-28 mortality in this subgroup was 53% (318/598). Mortality was 56% (168/300) in the hydrocortisone group and 50% (150/298) in the control group. In the multivariable analysis (N = 546), hydrocortisone use was not significantly associated with day-28 mortality in patients with septic shock (OR 1.392; 95% CI 0.952–2.036; p = 0.088) (Fig 2 and Table 2).

Sepsis without shock was documented in 444 matched patients. Day-28 mortality in this subgroup was 36% (162/444). Mortality was 42% (92/221) in the hydrocortisone group and 31% (70/223) in the control group. This association persisted in the multivariable analysis (N = 389) (OR 1.697; 95% CI 1.051–2.740; p = 0.031) (Fig 2 and Table 2).

The matched cohort included 368 female patients, with day-28 mortality of 48% (177/368). Mortality was 54% (97/181) in the hydrocortisone group and 43% (80/187) in the control group. In the multivariable analysis (N = 329), hydrocortisone use was associated with day-28 mortality in the female subgroup (OR 1.718; 95% CI 1.055–2.796; p = 0.030) (Fig 2 and Table 2). However, the formal hydrocortisone × sex interaction was not statistically significant; therefore, this subgroup finding should be considered exploratory.

In contrast, hydrocortisone use was not associated with day-28 mortality among patients receiving norepinephrine-equivalent dose ≥0.25 µg/kg/min at HA-BSI diagnosis (S8 Table).

### Sensitivity analysis

**Fig 3:**
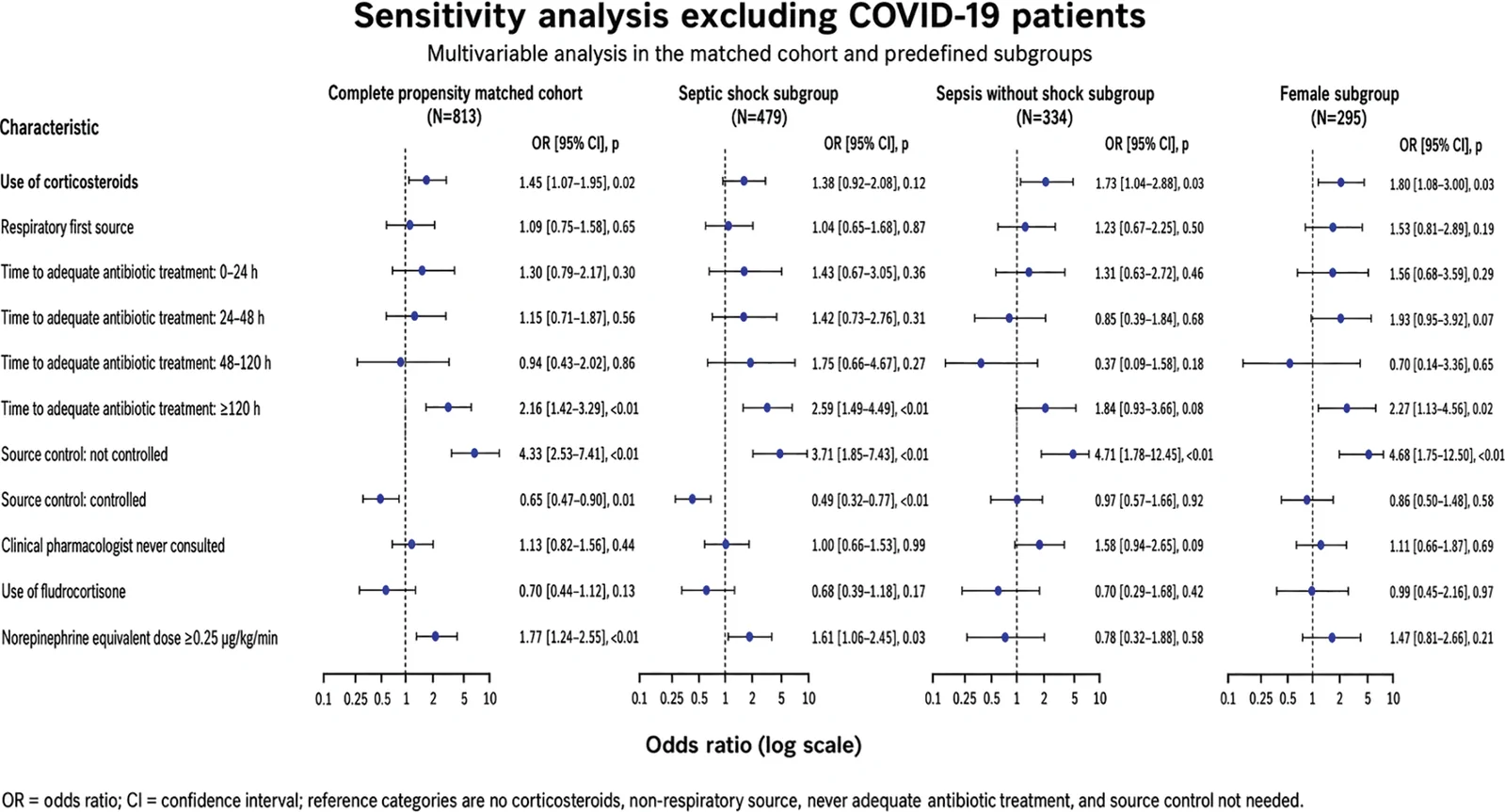
Forest plot of the multivariable analysis after exclusion of patients admitted for COVID-19.

A sensitivity analysis excluding patients admitted to the ICU for COVID-19 included 813 patients in the complete-case multivariable model. In this analysis, hydrocortisone use remained associated with increased day-28 mortality in the overall matched cohort (OR 1.445; 95% CI 1.072–1.947; p = 0.016), whereas no significant association was observed among patients with septic shock or high-dose norepinephrine (Fig 3).

**Fig 4:**
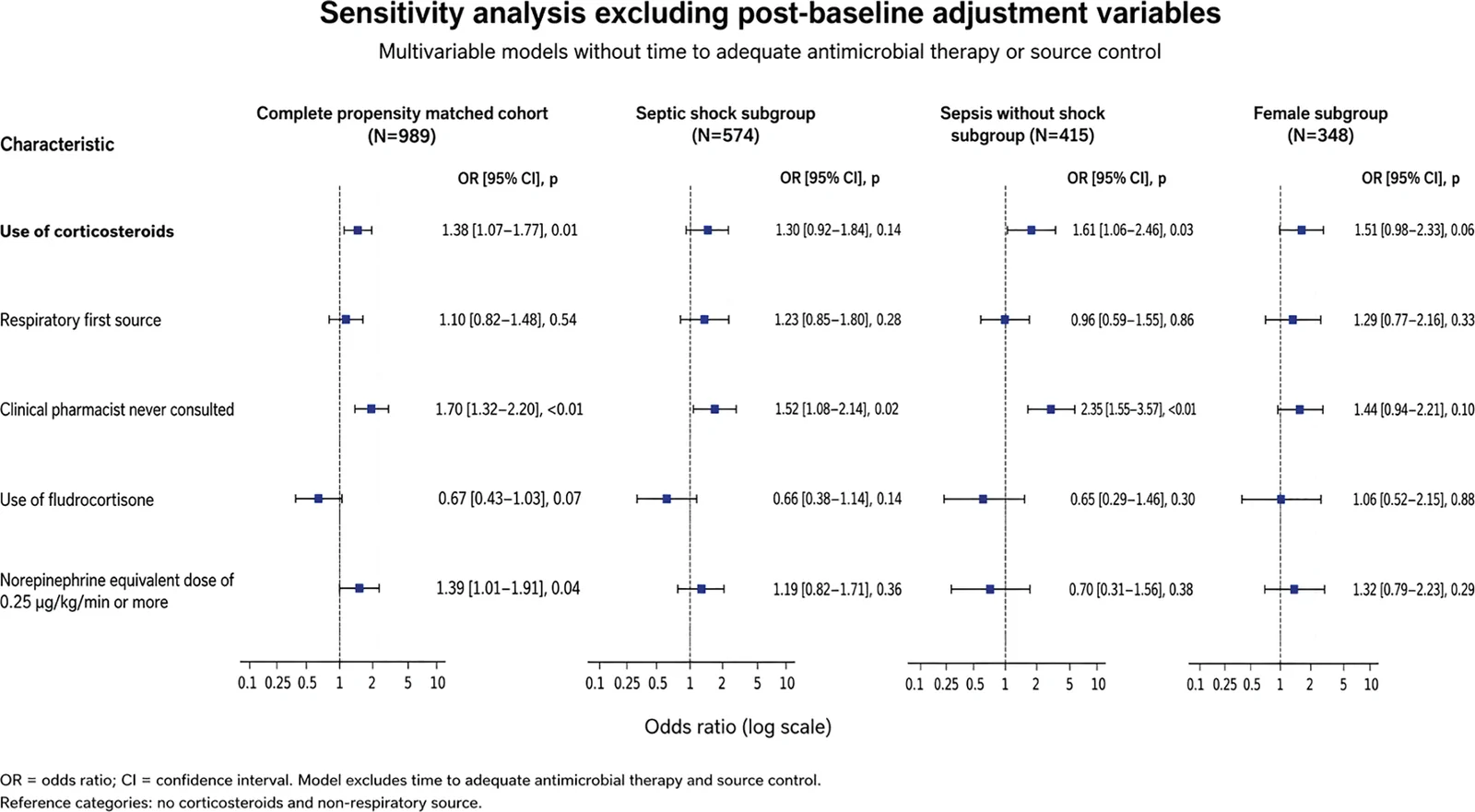
Forest plot of the sensitivity analysis excluding time to adequate antimicrobial therapy and source control from the multivariable model.

**Fig 5:**
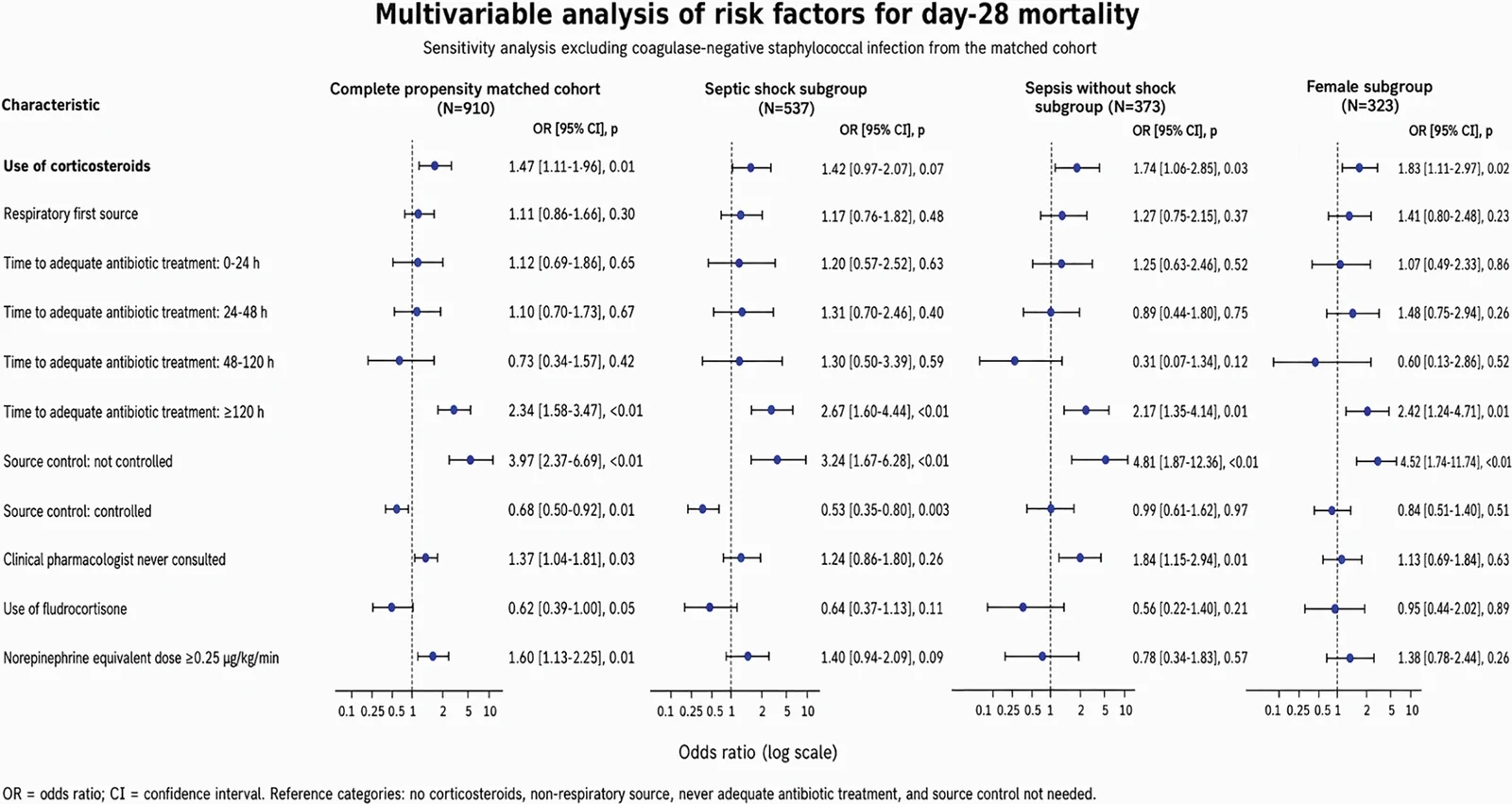
Forest plot of the sensitivity analysis excluding coagulase-negative staphylococcal bloodstream infections from the matched cohort.

Additional sensitivity analyses are presented in Figs 4 and 5 and in the Supplementary Materials. When time to adequate antimicrobial therapy and source control were removed from the multivariable model, the association between hydrocortisone use and day-28 mortality persisted in the overall matched cohort, in patients with sepsis without shock, and female subgroup, but not in patients with septic shock or high-dose norepinephrine. After exclusion of patients with coagulase-negative staphylococcal bloodstream infection from the already matched cohort, the overall association also persisted, while no significant association was observed in patients with septic shock or high-dose norepinephrine.

### Secondary outcomes

Exploratory secondary outcomes are presented in Table 3. Hydrocortisone exposure was associated with fewer vasopressor-free days and fewer mechanical ventilation-free days at day 28, and with a less favorable SOFA trajectory between HA-BSI diagnosis and day 7. These secondary analyses were exploratory and should be interpreted cautiously because of the observational design and the post-baseline nature of these outcomes.

**Table 3:** Exploratory analysis of secondary outcomes in the propensity-matched cohort. Each outcome was analyzed using a linear regression model with cluster-robust standard errors by matched pair, adjusted for the same covariates as the main multivariable model. Estimates are adjusted mean differences (β).

Characteristic	Vasopressor-free days at day 28 (N = 935)			Mechanical ventilation-free days at day 28 (N = 935)			SOFA variation from BSI to day 7 (N = 656)		
	β	95% CI	p	β	95% CI	p	β	95% CI	p
Hydrocortisone exposure	−3.15	−4.60; −1.71	<0.001	−2.31	−3.73; −0.89	0.001	0.97	0.39; 1.55	0.001
Respiratory first source	−1.17	−2.95; 0.61	0.199	−2.57	−4.28; −0.87	0.003	0.13	−0.56; 0.82	0.703
**Time to adequate antimicrobial therapy**									
Never (reference)	—	—	—	—	—	—	—	—	—
0–24 h	−0.04	−2.47; 2.39	0.973	−0.58	−2.98; 1.82	0.638	0.31	−0.70; 1.32	0.549
24–48 h	−2.22	−4.57; 0.13	0.064	−3.23	−5.26; −1.20	0.002	0.88	0.11; 1.66	0.026
48–120 h	−0.32	−4.20; 3.57	0.872	−1.55	−5.34; 2.23	0.422	0.85	−0.57; 2.28	0.241
≥120 h	−4.23	−6.23; −2.23	<0.001	−4.06	−5.87; −2.24	<0.001	0.68	−0.27; 1.63	0.159
**Source control**									
Not needed (reference)	—	—	—	—	—	—	—	—	—
Not controlled	−7.14	−9.25; −5.04	<0.001	−6.67	−8.58; −4.75	<0.001	1.45	0.16; 2.74	0.028
Controlled	1.67	0.00; 3.35	0.050	0.56	−1.07; 2.18	0.501	−0.30	−0.92; 0.31	0.334
Clinical pharmacist never consulted	−2.83	−4.31; −1.35	<0.001	−2.91	−4.36; −1.46	<0.001	0.72	0.13; 1.32	0.017
Centre use of fludrocortisone	1.54	−0.80; 3.87	0.197	2.32	−0.05; 4.70	0.055	−0.26	−1.25; 0.73	0.601
Norepinephrine equivalent dose ≥0.25 µg/kg/min	−2.42	−4.20; −0.64	0.008	−2.47	−4.12; −0.83	0.003	−1.43	−2.17; −0.69	<0.001

β = adjusted mean difference; CI = confidence interval; BSI = bloodstream infection. For time to adequate antimicrobial therapy, the reference category is ‘never’. For source control, the reference category is ‘not needed’. For free-day outcomes, negative β values indicate fewer days alive and free of the support. For SOFA variation, positive β values indicate higher recorded delta_SOFA values with SOFA values increasing between BSI day and day 7. These analyses are exploratory and should not be interpreted causally.

## Discussion

To the best of our knowledge, this is the first study to assess the association between hydrocortisone use and day-28 mortality in ICU patients with sepsis and HA-BSI. In the propensity-matched cohort, hydrocortisone use was associated with increased day-28 mortality in the overall population. This association persisted after excluding patients admitted for COVID-19, after excluded potential confounding factor regarding the quality of post exposure treatment and after excluding patients with coagulase-negative staphylococcal bloodstream infection. However, no significant association was observed among patients with septic shock or among those receiving norepinephrine-equivalent dose ≥0.25 µg/kg/min. These findings suggest that the observed signal was mainly driven by patients outside the subgroup most consistent with current guideline-based hydrocortisone indications.

A strength of this study is that it assesses real-life hydrocortisone use in a large international cohort of patients with microbiologically documented HA-BSI. Unlike several corticosteroid trials in sepsis or severe infections, inclusion in EUROBACT-2 required a positive blood culture, ensuring microbiological documentation of the infectious episode [6,7]. This setting allowed us to explore hydrocortisone use in a specific hospital-acquired proved infection context that differs from many randomized trials conducted in broader septic shock or respiratory infection populations where the microbiological documentation vary between 55 and 72% [2–4,6].

Our findings should be interpreted in the context of the heterogeneous literature on corticosteroids in sepsis. Recent guideline updates support hydrocortisone use in selected patients with septic shock, mainly to accelerate shock resolution, but the effect on survival remains less certain [1,13]. In our cohort, hydrocortisone was not associated with increased mortality among patients with septic shock or high-dose norepinephrine. By contrast, the association with mortality was observed mainly in patients without shock and in the low-dose norepinephrine subgroup. These observations are consistent with guidelines and the hypothesis that corticosteroid effects may vary according to sepsis phenotype and severity [13,14]. In a large, emulated target trial framework based on 4,000 patients and stratified by a machine learning method, Rajendran et al. found that the use of corticosteroids in critically ill patients with infection was associated with an increase in organ failure, notably among those having a mild severe infection and those showing rapid improvement [14]. Consequently, the association explored by this work align with the existing data that suggests corticosteroids may be deleterious in patients with less severe sepsis.

The association observed in female patients should be interpreted cautiously. Although sex-related differences in sepsis and immune response have been described [15], the deleterious hydrocortisone & sex association found in the female subgroup result should be considered exploratory and hypothesis-generating rather than evidence of a sex-specific treatment effect.

This study has several limitations. First, hydrocortisone use was not randomized, and the rationale for administration was not recorded; residual confounding by indication may persist despite propensity score matching and multivariable adjustment. Second, the exact date and time of hydrocortisone initiation relative to blood culture sampling or HA-BSI diagnosis were not available, and time-dependent bias cannot be excluded. Third, daily patient-level hydrocortisone dosing was not available, and centre-level practices differed regarding dose, duration, and association with fludrocortisone [16]. Fourth, exact same-centre or same-country matching was not feasible without major loss of sample size, and residual centre/country effects may persist. Fifth, propensity score matching restricted the analysis to patients within the region of common support; therefore, findings may not apply to the entire EUROBACT-2 HA-BSI population. Finally, HA-BSI patients remained heterogeneous in terms of severity, source of infection, microbiology, immune status, and inflammatory phenotype, which may influence corticosteroid response [16,17].

## Conclusion

Hydrocortisone use was associated with increased day-28 mortality in the overall propensity-matched cohort of ICU patients with HA-BSI. This association persisted in several sensitivity analyses but was not observed among patients with septic shock or high-dose norepinephrine. These findings should be interpreted cautiously as observational and hypothesis-generating, and they do not establish a causal harmful effect of hydrocortisone.

## Supporting information

S1 Table Full details of the cohort. (DOCX)

S2 Table Bloodstream infection information and physiological conditions at the time of blood culture sampling. (DOCX)

S3 Table Bacteriological characteristics of bloodstream infections. (DOCX)

S4 Table Centre characteristics. (DOCX)

S5 Table Corticosteroid practice by centre. (DOCX)

S6 Table Descriptive analysis of centres using fludrocortisone. (DOCX)

S7 Table Characteristics of matched and unmatched patients after propensity score matching. (DOCX)

S8 Table Multivariable analysis in the high-dose norepinephrine subgroup. (DOCX)

S1 Fig Directed acyclic graph. (TIF)

S2 Fig Balance for propensity score variables and patient-level multivariable covariates. (TIF)

S3 Fig Balance for centre, country, and income variables. (TIF)

S4 Fig Balance for relevant variables not included in the propensity score or multivariable model. (TIF)

S1 File EUROBACT2 investigators. (DOCX)
