# Supplementary material for: Association between hydrocortisone use and outcomes in sepsis patients with hospital-acquired bloodstream infections: An ancillary analysis of the EUROBACT-2 cohort
Source: PLoS One. 2026 Sep 15;21(9):e0356721. doi: 10.1371/journal.pone.0356721 (PMC13577328; doi:10.1371/journal.pone.0356721)
Supplement: Supplementary file 1 [file pone.0356721.s001.docx]

|  | **Supplemental table 1 : full details of the cohort** | | | | | | | |
| --- | --- | --- | --- | --- | --- | --- | --- | --- |
|  | **Before matching** | | | | **After matching** | | | |
|  | **Overall 2401 (100%)** | **Controls 1793 (74.6%)** | **Hydrocortisone 608 (23.4%)** | **p value** | **Overall 1042 (100%)** | **Controls 521 (50.0%)** | **Hydrocortisone 521 (50.0%)** | **p value** |
| Characteristics | | | | | | | | |
| Male gender | 1543 (64%) | 1161 (65%) | 382 (63%) | 0.4 | 674 (65%) | 334 (64%) | 340 (65%) | 0.7 |
| Age (years) | 61 (16) | 61 (16) | 62 (16) | 0.9 | 63 (16) | 63 (16) | 62 (16) | 0.8 |
| BMI | 27.4 (7.0) | 27.4 (7.1) | 27.4 (6.5) | 0.9 | 27.3 (6.4) | 27.0 (6.1) | 27.5 (6.6) | 0.08 |
| Previous health status | | | | | | | | |
| Prior Good Health | 1,053 (44%) | 816 (46%) | 237 (39%) | 0.011 | 421 (40%) | 212 (41%) | 209 (40%) | 0.4 |
| Moderate limitation | 826 (34%) | 587 (33%) | 239 (39%) |  | 384 (37%) | 185 (36%) | 199 (38%) |  |
| Serious limitation | 344 (14%) | 252 (14%) | 92 (15%) |  | 158 (15%) | 78 (15%) | 80 (15%) |  |
| Severe limitation | 177 (7.4%) | 137 (7.6%) | 40 (6.6%) |  | 78 (7.5%) | 45 (8.7%) | 33 (6.3%) |  |
| Charlson comorbidity score | 2.27 (2.57) | 2.20 (2.54) | 2.48 (2.67) | 0.007 | 2.50 (2.69) | 2.52 (2.68) | 2.48 (2.71) | 0.6 |
| Chronic disease | | | | | | | | |
| Moderate COPD | 288 (12%) | 206 (11%) | 82 (13%) | 0.2 | 133 (13%) | 66 (13%) | 67 (13%) | 0.5 |
| Severe COPD | 99 (4.1%) | 70 (3.9%) | 29 (4.8%) | 0.4 | 39 (3.7%) | 16 (3.1%) | 23 (4.4%) | 0.9 |
| Heart failure (NYHA 3) | 198 (8.4%) | 146 (8.4%) | 52 (8.4%) | 1 | 91 (8.7%) | 50 (9.6%) | 41 (7.9%) | 0.5 |
| Heart failure (NYHA 4) | 57 (2.4%) | 42 (2.4%) | 15 (2.4%) | 1 | 25 (2.4%) | 12 (2.3%) | 13 (2.5%) | 0.4 |
| Previous Myocardial infarction | 225 (9.4%) | 159 (8.9%) | 66 (11%) | 0.15 | 119 (11%) | 66 (13%) | 53 (10%) | 0.7 |
| Peripherical vascular disease | 161 (6.7%) | 123 (6.9%) | 38 (6.3%) | 0.6 | 76 (7.3%) | 45 (8.6%) | 31 (6.0%) | 1 |
| Cerebro-vascular disease | 263 (11%) | 214 (12%) | 49 (8.1%) | 0.008 | 106 (10%) | 63 (12%) | 43 (8.3%) | 0.5 |
| Dementia | 99 (4.1%) | 75 (4.2%) | 24 (3.9%) | 0.8 | 46 (4.4%) | 25 (4.8%) | 21 (4.0%) | 0.4 |
| Hemiplegia | 62 (2.6%) | 43 (2.4%) | 19 (3.1%) | 0.3 | 28 (2.7%) | 13 (2.5%) | 15 (2.9%) | 0.3 |
| Diabetes without end organ damages | 439 (18%) | 307 (17%) | 132 (22%) | 0.011 | 195 (19%) | 93 (18%) | 102 (20%) | 0.2 |
| Diabetes with end organ damages | 251 (10%) | 184 (10%) | 67 (11%) | 0.6 | 121 (12%) | 59 (11%) | 62 (12%) | 0.4 |
| Moderate renal disease | 233 (9.7%) | 164 (9.1%) | 69 (11%) | 0.11 | 124 (12%) | 60 (12%) | 64 (12%) | 1 |
| Reanal disease with chronic dialysis | 115 (4.8%) | 83 (4.6%) | 32 (5.3%) | 0.5 | 55 (5.3%) | 29 (5.6%) | 26 (5.0%) | 0.4 |
| Connective tissue disease | 39 (1.6%) | 27 (1.5%) | 12 (2.0%) | 0.4 | 22 (2.1%) | 10 (1.9%) | 12 (2.3%) | 0.5 |
| Gastro-duodenal ulcer disease | 80 (3.3%) | 59 (3.3%) | 21 (3.5%) | 0.8 | 35 (3.4%) | 17 (3.3%) | 18 (3.5%) | 1 |
| Mild to moderate liver disease | 82 (3.4%) | 59 (3.3%) | 23 (3.8%) | 0.6 | 38 (3.6%) | 17 (3.3%) | 21 (4.0%) | 0.6 |
| Severe liver disease | 62 (2.6%) | 44 (2.5%) | 18 (3.0%) | 0.5 | 31 (3.0%) | 14 (2.7%) | 17 (3.3%) | 0.7 |
| Chemotherapy or radiotheraphy within 6 months | 190 (7.9%) | 137 (7.6%) | 53 (8.7%) | 0.4 | 96 (9.2%) | 46 (8.8%) | 50 (9.6%) | 1 |
| Organ transplant | 58 (2.4%) | 41 (2.3%) | 17 (2.8%) | 0.5 | 29 (2.8%) | 14 (2.7%) | 15 (2.9%) | 0.9 |
| AIDS | 15 (0.6%) | 12 (0.7%) | 3 (0.5%) | 0.8 | 4 (0.4%) | 2 (0.4%) | 2 (0.4%) | 0.7 |
| Other immunodepression | 71 (3.0%) | 53 (3.0%) | 18 (3.0%) | 1 | 29 (2.8%) | 13 (2.5%) | 16 (3.1%) | 0.3 |
| Solid tumors without metastasis | 228 (9.5%) | 170 (9.5%) | 58 (9.6%) | 1 | 36 (3.5%) | 19 (3.6%) | 17 (3.3%) | 0.5 |
| Haematological malignancy | 129 (5.4%) | 78 (4.4%) | 51 (8.4%) | < 0.001 | 82 (7.9%) | 37 (7.1%) | 45 (8.6%) | 0.6 |
| Source of ICU admission | | | | | | | | |
| Other hospital | 331 (14%) | 263 (15%) | 68 (11%) | 0.002 | 126 (12%) | 67 (13%) | 59 (11%) | 0.01 |
| Emergency department | 714 (30%) | 525 (29%) | 189 (31%) |  | 289 (28%) | 134 (26%) | 155 (30%) |  |
| Operating room | 271 (11%) | 225 (13%) | 46 (7.6%) |  | 106 (10%) | 62 (12%) | 44 (8.4%) |  |
| Hospital ward | 995 (41%) | 713 (40%) | 282 (46%) |  | 487 (47%) | 245 (47%) | 242 (46%) |  |
| Other intermediate or intensive care unit | 64 (2.7%) | 47 (2.6%) | 17 (2.8%) |  | 25 (2.4%) | 10 (1.9%) | 15 (2.9%) |  |
| Other | 26 (1.1%) | 20 (1.1%) | 6 (1.0%) |  | 9 (0.9%) | 3 (0.6%) | 6 (1.2%) |  |

***BMI: Body mass index; COPD: Chronic obstructive pulmonary disease; NYHA: New York Heart Association; AIDS : Acquired immunodeficiency syndrome.***
