# Supplementary material for: Association between hydrocortisone use and outcomes in sepsis patients with hospital-acquired bloodstream infections: An ancillary analysis of the EUROBACT-2 cohort
Source: PLoS One. 2026 Sep 15;21(9):e0356721. doi: 10.1371/journal.pone.0356721 (PMC13577328; doi:10.1371/journal.pone.0356721)
Supplement: Supplementary file 2 [file pone.0356721.s002.docx]

**Supplemental Table 2: Blood stream infection informations and physiological conditions at the time of blood stream sampling**

|  | **Before matching** | | | | **After matching** | | | |
| --- | --- | --- | --- | --- | --- | --- | --- | --- |
|  | **Overall 2401 (100%)** | **Controls 1793 (74.6%)** | **Hydrocortisone 608 (25.4%)** | **p value** | **Overall 1042 (100%)** | **Controls 521 (50.0%)** | **Hydrocortisone 521 (50.0%)** | **p value** |
| **Bloodstream infection information** | | | | | | | | |
| Time of BSI sampling | | | | | | | | |
| Before ICU admission | 500 (21%) | 345 (19%) | 155 (26%) | <0.001 | 258 (25%) | 124 (24%) | 134 (26%) | 0.4 |
| Less than 2 days after ICU admission | 210 (8.8%) | 141 (7.9%) | 69 (11%) |  | 125 (12%) | 62 (12%) | 63 (12%) |  |
| More than 2 days after ICU admission | 1.685 (70%) | 1.304 (73%) | 381 (63%) |  | 659 (63%) | 335 (64%) | 324 (62%) |  |
| Delay between hospital admission and BS sampling (days) | 18 (30) | 21 (35) | 17 (22) | <0.001 | 19.0 (26) | 22 (29) | 17 (23) | 0.008 |
| Time of positivity of BS (hours) | 32 (31) | 33 (33) | 31 (29) | 0.2 | 31 (30) | 31 (30) | 31 (30) | 0.9 |
| 0-24 | 926 (39%) | 698 (39%) | 228 (38%) | 0.5 | 419 (40%) | 207(39%) | 212 (40%) | 0.4 |
| 24-48 | 288 (12%) | 233 (13%) | 68 (11%) |  | 128(12%) | 69 (13%) | 59 (12%) |  |
| 48-120 | 384 (16%) | 287 (16%) | 79 (13%) |  | 138 (13%) | 69 (13%) | 69 (13%) |  |
| 120+ | 120 (5%) | 90 (5%) | 25 (4%) |  | 48 (4%) | 27 (5%) | 21 (4 %) |  |
| Missing | 693 (29%) | 485 (27%) | 208 (34%) |  | 331 (31%) | 160 (30%) | 171 (32%) |  |
| Physiological conditions at the time of bloodstream sampling | | | | | | | | |
| SOFA respiratory component | 2.13 (1.31) | 2.02 (1.32) | 2.45 (1.23) | <0.001 | 2.4 (1.3) | 2.3 (1.3) | 2.4 (1.2) | 0.6 |
| SOFA hematological component | 0.75 (1.14) | 0.64 (1.07) | 1.06 (1.28) | <0.001 | 1.0 (1.2) | 0.9 (1.2) | 1.0 (1.3) | 0.2 |
| SOFA cardio vascular component | 2.17 (1.71) | 1.89 (1.70) | 3.00 (1.48) | <0.001 | 2.9 (1.5) | 2.8 (1.5) | 3.0 (1.5) | <0.001 |
| SOFA neurologic component | 1.66 (1.50) | 1.60 (1.49) | 1.82 (1.50) | 0.029 | 1.7 (1.5) | 1.7 (1.6) | 1.7 (1.5) | 0.6 |
| SOFA hepatic component | 0.71 (1.05) | 0.66 (1.03) | 0.86 (1.12) | <0.001 | 0.9 (1.1) | 0.9 (1.1) | 0.9 (1.1) | 0.2 |
| SOFA renal component | 1.23 (1.49) | 1.12 (1.46) | 1.56 (1.54) | <0.001 | 1.5 (1.5) | 1.5 (1.5) | 1.6 (1.6) | 1 |
| SOFA global | 8.4 (4.3) | 7.7 (4.1) | 10.5 (4.1) | <0.001 | 10.2 (4.1) | 10.0 (4.1) | 10.5 (4.1) | 0.1 |
| pH | 7.35 (0.13) | 7.36 (0.11) | 7.31 (0.15) | <0.001 | 7.32 (0.14) | 7.34 (0.12) | 7.31 (0.15) | 0.022 |
| Maximum lactatemia (mmol/L) | 2.94 (3.13) | 2.55 (2.66) | 4.10 (3.99) | <0.001 | 3.9 (3.9) | 3.8 (3.8) | 4.0 (4.0) | 0.05 |
| Renal replacement use | 473 (20%) | 316 (18%) | 157 (26%) | <0.001 | 264 (25%) | 124 (24%) | 140 (27%) | 0.11 |
| ECMO use | 32 (1%) | 17 (1%) | 15 (2%) | 0.013 | 27 (2.6%) | 12 (2.3%) | 15 (2.9%) | 0.07 |
| Ventilation status | | | | | | | | |
| Invasive mechanical ventilation | 1.666 (69%) | 1.189 (66%) | 477 (78%) | <0.001 | 788 (76%) | 373 (72%) | 415 (80%) | 0.1 |
| Low flow oxygen or no oxygen | 453 (19%) | 385 (21%) | 68 (11%) |  | 154 (15%) | 95 (18%) | 59 (11%) |  |
| High flow oxygen | 148 (6.2%) | 114 (6.4%) | 34 (5.6%) |  | 52 (5.0%) | 26 (5.0%) | 26 (5.0%) |  |
| Non-invasive mechanical ventilation or CPAP | 134 (5.6%) | 105 (5.9%) | 29 (4.8%) |  | 48 (4.6%) | 27 (5.2%) | 21 (4.0%) |  |
| ICU: Intensive care unit; BSI: Bloodstream infection; SOFA: sequential organ failure assessment; ECMO: Extracorporeal membrane oxygenation; CPAP: Continuous positive airway pressure. | | | | | | | | |
