# Supplementary material for: Association between hydrocortisone use and outcomes in sepsis patients with hospital-acquired bloodstream infections: An ancillary analysis of the EUROBACT-2 cohort
Source: PLoS One. 2026 Sep 15;21(9):e0356721. doi: 10.1371/journal.pone.0356721 (PMC13577328; doi:10.1371/journal.pone.0356721)
Supplement: Supplementary file 3 [file pone.0356721.s003.docx]

**Supplemental Table 3: Bacteriological characteristics of bloodstream infections.**

|  | **Before matching** | | | | **After matching** | | | |
| --- | --- | --- | --- | --- | --- | --- | --- | --- |
|  | **Overall 2401 (100%)** | **Controls 1793 (74.6%)** | **Hydrocortisone 608(23.4%)** | **p value** | **Overall 1042 (100%)** | **Controls 521 (50.0%)** | **Hydrocortisone 521 (50.0%)** | **p value** |
| **Pathogen documentation** | | | | | | | | |
| Gram negative pathogen | 1488 (62%) | 1079 (60%) | 409 (67%) | 0.002 | 677 (65%) | 328 (63%) | 349 (67%) | 0.13 |
| Gram positive pathogen | 800 (33%) | 629 (35%) | 171 (28%) | 0.002 | 318 (31%) | 170 (33%) | 148 (28%) | 0.042 |
| Anaerobic pathogen | 56 (2.3%) | 43 (2.4%) | 13 (2.1%) | 0.7 | 21 (2.0%) | 9 (1.7%) | 12 (2.3%) | 0.3 |
| Difficult to treat gram negative pathogen | 320 (23%) | 212 (21%) | 108 (28%) | 0.006 | 169 (26%) | 76 (25%) | 93 (28%) | 0.2 |
| Any resistant pathogen | 598 (25%) | 434 (24%) | 164 (27%) | 0.2 | 265 (25%) | 129 (25%) | 136 (26%) | 1 |
