# Supplementary material for: Association between hydrocortisone use and outcomes in sepsis patients with hospital-acquired bloodstream infections: An ancillary analysis of the EUROBACT-2 cohort
Source: PLoS One. 2026 Sep 15;21(9):e0356721. doi: 10.1371/journal.pone.0356721 (PMC13577328; doi:10.1371/journal.pone.0356721)
Supplement: Supplementary file 4 [file pone.0356721.s004.docx]

**Supplemental Table 4 : Centers characteristics**

|  | **Before matching** | | | | **After matching** | | | |
| --- | --- | --- | --- | --- | --- | --- | --- | --- |
|  | **Overall 2401 (100%)** | **Controls 1793 (74.6%)** | **Hydrocortisone 608 (23.4%)** | **p value** | **Overall 1042 (100%)** | **Controls 521 (50.0%)** | **Hydrocortisone 521 (50.0%)** | **p value** |
| **Specialist availability** | | | | | | | | |
| No infectious specialist available | 138 (5.8%) | 101 (5.7%) | 37 (6.1%) | 0.7 | 70 (6.6%) | 36 (6.8%) | 34 (6.4%) | 0.8 |
| No clinical pharmacologist available | 1010 (42%) | 756 (42%) | 254 (42%) | 0.8 | 431 (42%) | 219 (42%) | 212 (41%) | 0.5 |
| Income | | | | | | | | |
| High-income | 1376 (57%) | 1049 (59%) | 327 (54%) | <0.001 | 613 (59%) | 309 (59%) | 304 (58%) | 0.8 |
| Upper-middle-income | 787 (33%) | 600 (33%) | 187 (31%) |  | 306 (29%) | 153 (29%) | 153 (29%) |  |
| Lower-middle-income | 227 (9.5%) | 139 (7.8%) | 88 (14%) |  | 116 (11%) | 56 (11%) | 60 (12%) |  |
| Low-income | 11 (0.5%) | 5 (0.3%) | 6 (1.0%) |  | 7 (0.7%) | 3 (0.6%) | 4 (0.8%) |  |
| Center Country | | | | | | | | |
| Argentina | 23 (1.0%) | 20 (1.1%) | 3 (0.5%) | <0.001 | 4 (0.4%) | 1 (0.2%) | 3 (0.6%) | 1 |
| Australia | 95 (4.0%) | 64 (3.6%) | 31 (5.1%) |  | 63 (6.0%) | 33 (6.3%) | 30 (5.8%) |  |
| Bangladesh | 6 (0.2%) | 1 (<0.1%) | 5 (0.8%) |  | 2 (0.2%) | 1 (0.2%) | 1 (0.2%) |  |
| Belgium | 57 (2.4%) | 50 (2.8%) | 7 (1.2%) |  | 16 (1.5%) | 9 (1.7%) | 7 (1.3%) |  |
| Brunei | 27 (1.1%) | 17 (0.9%) | 10 (1.6%) |  | 14 (1.3%) | 7 (1.3%) | 7 (1.3%) |  |
| Canada | 18 (0.7%) | 14 (0.8%) | 4 (0.7%) |  | 10 (1.0%) | 6 (1.2%) | 4 (0.8%) |  |
| China | 76 (3.2%) | 61 (3.4%) | 15 (2.5%) |  | 29 (2.8%) | 16 (3.1%) | 13 (2.5%) |  |
| Colombia | 7 (0.3%) | 6 (0.3%) | 1 (0.2%) |  | 1 (<0.1%) | 0 (0.0%) | 1 (0.2%) |  |
| Croatia | 6 (0.2%) | 6 (0.3%) | 0 (0.0%) |  | 0 (0.0%) | 0 (0.0%) | 0 (0.0%) |  |
| Dubai | 8 (0.3%) | 7 (0.4%) | 1 (0.2%) |  | 1 (<0.1%) | 0 (0.0%) | 1 (0.2%) |  |
| Egypt | 36 (1.5%) | 20 (1.1%) | 16 (2.6%) |  | 27 (2.6%) | 12 (2.3%) | 15 (2.9%) |  |
| France | 269 (11%) | 213 (12%) | 56 (9.2%) |  | 98 (9.4%) | 46 (8.8%) | 52 (10.0%) |  |
| Germany | 44 (1.8%) | 36 (2.0%) | 8 (1.3%) |  | 15 (1.4%) | 7 (1.3%) | 8 (1.5%) |  |
| Greece | 132 (5.5%) | 84 (4.7%) | 48 (7.9%) |  | 85 (8.2%) | 42 (8.1%) | 43 (8.3%) |  |
| Hong Kong | 5 (0.2%) | 3 (0.2%) | 2 (0.3%) |  | 4 (0.4%) | 2 (0.4%) | 2 (0.4%) |  |
| India | 47 (2.0%) | 27 (1.5%) | 20 (3.3%) |  | 37 (3.6%) | 19 (3.6%) | 18 (3.5%) |  |
| Iran | 47 (2.0%) | 27 (1.5%) | 20 (3.3%) |  | 6 (0.6%) | 3 (0.6%) | 3 (0.6%) |  |
| Iraq | 2 (<0.1%) | 0 (0.0%) | 2 (0.3%) |  | 0 (0.0%) | 0 (0.0%) | 0 (0.0%) |  |
| Israel | 18 (0.7%) | 11 (0.6%) | 7 (1.2%) |  | 12 (1.2%) | 6 (1.2%) | 6 (1.2%) |  |
| Italy | 147 (6.1%) | 115 (6.4%) | 32 (5.3%) |  | 65 (6.2%) | 33 (6.3%) | 32 (6.1%) |  |
| Japan | 40 (1.7%) | 30 (1.7%) | 10 (1.6%) |  | 21 (2.0%) | 11 (2.1%) | 10 (1.9%) |  |
| Kazakhstan | 7 (0.3%) | 7 (0.4%) | 0 (0.0%) |  | 0 (0.0%) | 0 (0.0%) | 0 (0.0%) |  |
| Lebanon | 7 (0.3%) | 4 (0.2%) | 3 (0.5%) |  | 3 (0.3%) | 2 (0.4%) | 1 (0.2%) |  |
| Libya | 20 (0.8%) | 8 (0.4%) | 12 (2.0%) |  | 12 (1.2%) | 5 (1.0%) | 7 (1.3%) |  |
| Malaysia | 36 (1.5%) | 30 (1.7%) | 6 (1.0%) |  | 11 (1.1%) | 6 (1.2%) | 5 (1.0%) |  |
| Mexico | 18 (0.7%) | 6 (0.3%) | 12 (2.0%) |  | 15 (1.4%) | 6 (1.2%) | 9 (1.7%) |  |
| Morocco | 46 (1.9%) | 34 (1.9%) | 12 (2.0%) |  | 17 (1.6%) | 8 (1.5%) | 9 (1.7%) |  |
| Nigeria | 5 (0.2%) | 5 (0.3%) | 0 (0.0%) |  | 0 (0.0%) | 0 (0.0%) | 0 (0.0%) |  |
| Philippines | 8 (0.3%) | 5 (0.3%) | 3 (0.5%) |  | 4 (0.4%) | 1 (0.2%) | 3 (0.6%) |  |
| Poland | 40 (1.7%) | 27 (1.5%) | 13 (2.1%) |  | 25 (2.4%) | 12 (2.3%) | 13 (2.5%) |  |
| Portugal | 71 (3.0%) | 54 (3.0%) | 17 (2.8%) |  | 35 (3.4%) | 18 (3.5%) | 17 (3.3%) |  |
| Qatar | 15 (0.6%) | 10 (0.6%) | 5 (0.8%) |  | 12 (1.2%) | 7 (1.3%) | 5 (1.0%) |  |
| Republic of Ireland | 7 (0.3%) | 6 (0.3%) | 1 (0.2%) |  | 2 (0.2%) | 1 (0.2%) | 1 (0.2%) |  |
| Republic of Korea | 33 (1.4%) | 26 (1.5%) | 7 (1.2%) |  | 15 (1.4%) | 8 (1.5%) | 7 (1.3%) |  |
| Romania | 34 (1.4%) | 22 (1.2%) | 12 (2.0%) |  | 22 (2.1%) | 10 (1.9%) | 12 (2.3%) |  |
| Russian Federation | 40 (1.7%) | 40 (2.2%) | 0 (0.0%) |  | 0 (0.0%) | 0 (0.0%) | 0 (0.0%) |  |
| Saudi Arabia | 14 (0.6%) | 7 (0.4%) | 7 (1.2%) |  | 7 (0.7%) | 2 (0.4%) | 5 (1.0%) |  |
| Singapore | 16 (0.7%) | 8 (0.4%) | 8 (1.3%) |  | 11 (1.1%) | 6 (1.2%) | 5 (1.0%) |  |
| South Africa | 6 (0.2%) | 4 (0.2%) | 2 (0.3%) |  | 3 (0.3%) | 1 (0.2%) | 2 (0.4%) |  |
| Spain | 86 (3.6%) | 68 (3.8%) | 18 (3.0%) |  | 34 (3.3%) | 16 (3.1%) | 18 (3.5%) |  |
| Sudan | 9 (0.4%) | 4 (0.2%) | 5 (0.8%) |  | 5 (0.5%) | 2 (0.4%) | 3 (0.6%) |  |
| Sweden | 9 (0.4%) | 8 (0.4%) | 1 (0.2%) |  | 1 (<0.1%) | 0 (0.0%) | 1 (0.2%) |  |
| Switzerland | 18 (0.7%) | 15 (0.8%) | 3 (0.5%) |  | 5 (0.5%) | 2 (0.4%) | 3 (0.6%) |  |
| Syria | 2 (<0.1%) | 1 (<0.1%) | 1 (0.2%) |  | 2 (0.2%) | 1 (0.2%) | 1 (0.2%) |  |
| Taiwan | 35 (1.5%) | 25 (1.4%) | 10 (1.6%) |  | 12 (1.2%) | 6 (1.2%) | 6 (1.2%) |  |
| Thailand | 16 (0.7%) | 5 (0.3%) | 11 (1.8%) |  | 13 (1.2%) | 4 (0.8%) | 9 (1.7%) |  |
| Tunisia | 32 (1.3%) | 20 (1.1%) | 12 (2.0%) |  | 23 (2.2%) | 12 (2.3%) | 11 (2.1%) |  |
| Turkey | 483 (20%) | 383 (21%) | 100 (16%) |  | 183 (18%) | 98 (19%) | 85 (16%) |  |
| UK | 166 (6.9%) | 145 (8.1%) | 21 (3.5%) |  | 50 (4.8%) | 29 (5.6%) | 21 (4.0%) |  |
| bosnia and herzegovina | 10 (0.4%) | 3 (0.2%) | 7 (1.2%) |  | 8 (0.8%) | 3 (0.6%) | 5 (1.0%) |  |
| jordan | 2 (<0.1%) | 1 (<0.1%) | 1 (0.2%) |  | 2 (0.2%) | 1 (0.2%) | 1 (0.2%) |  |

*UK : United Kingdom .*
