# Supplementary material for: Association between hydrocortisone use and outcomes in sepsis patients with hospital-acquired bloodstream infections: An ancillary analysis of the EUROBACT-2 cohort
Source: PLoS One. 2026 Sep 15;21(9):e0356721. doi: 10.1371/journal.pone.0356721 (PMC13577328; doi:10.1371/journal.pone.0356721)
Supplement: Supplementary file 5 [file pone.0356721.s005.docx]

**Supplemental table 5: Corticosteroids practice description**

|  | **Before matching** | | | | **After matching** | | | |
| --- | --- | --- | --- | --- | --- | --- | --- | --- |
|  | **Overall 2401 (100%)** | **Controls 1793 (73.7%)** | **Hydrocortisone  608 (26.2%)** | **p value** | **Overall 1042 (100%)** | **Controls 521 (50.0%)** | **Hydrocortisone 521 (50.0%)** | **p value** |
| **Hydrocortisone practice in the inclusion center** | | | | | | | | |
| Septic shock | 840 (35%) | 573 (32%) | 261 (43%) | <0.001 |  | | | |
| Sepsis | 48 (2%) | 35 (2%) | 18 (3%) |  |  |  |  |  |
| Septic shock and high vasopressor | 1201 (50%) | 898 (50%) | 304 (50%) |  |  |  |  |  |
| Never | 312 (13%) | 287 (16%) | 25 (4%) |  |  |  |  |  |
| Dose of hydrocortisone used in the inclusion center | | | | | | | | |
| 50mg of hydrocortisone per 6 hours | 1,141 (53%) | 834 (53%) | 307 (54%) | 0.064 | 516 (53%) | 245 (51%) | 271 (55%) | 0.2 |
| 200mg of hydrocortisone in continuous infusion | 704 (33%) | 506 (32%) | 198 (35%) |  | 345 (35%) | 169 (35%) | 176 (36%) |  |
| Other | 290 (14%) | 229 (15%) | 61 (11%) |  | 114 (12%) | 69 (14%) | 45 (9.1%) |  |
| Duration of Hydrocortisone use in the inclusion center | | | | | | | | |
| Other | 183 (9.3%) | 138 (9.8%) | 45 (7.9%) | 0.026 |  | | | |
| 7 days | 344 (17.5%) | 226 (16.1%) | 118 (20.9%) |  |  |  |  |  |
| Until shock resolution | 1439 (73.1%) | 1038 (74.0%) | 401 (71.1%) |  |  |  |  |  |
| Administration per patients | | | | | | | | |
| Fludrocortisone administered | 86 (3.6%) | 0 (0%) | 86 (15%) | <0.001 | 110 (11%) | 38 (7.8%) | 72 (14%) | <0.001 |
| Hydrocortisone administration (in days) | 1.46 (3.36) | 0.00 (0.00) | 5.75 (4.48) | <0.001 | 5.6 (4.4) | 0 | 5.6 (4.4) | <0.001 |
