# Supplementary material for: Association between hydrocortisone use and outcomes in sepsis patients with hospital-acquired bloodstream infections: An ancillary analysis of the EUROBACT-2 cohort
Source: PLoS One. 2026 Sep 15;21(9):e0356721. doi: 10.1371/journal.pone.0356721 (PMC13577328; doi:10.1371/journal.pone.0356721)
Supplement: Supplementary file 6 [file pone.0356721.s006.docx]

**Supplemental TABLE 6: Descriptive analysis of centers using fludrocortisone**

Descriptive analysis on centers using fludrocortisone with hydrocortisone, including country distribution, steroid-treatment duration, and crude outcome description.

**Table A. Counts by fludrocortisone-centre status**

| **Level** | **Fludrocortisone-using centers/status** | **Non-fludrocortisone centers/status** | **Missing** |
| --- | --- | --- | --- |
| Center level | 29 | 298 | 27 |
| Patient level | 209 | 1945 | 247 |
| Hydrocortisone-treated patient level | 86 | 493 | 29 |

**Table B. Countries represented among fludrocortisone-using centres**

| **Country** | **Centres** | **Patients** | **Hydrocortisone patients** | **Deaths** | **Crude mortality (%)** | **Hydrocortisone use (%)** |
| --- | --- | --- | --- | --- | --- | --- |
| France | 17 | 118 | 32 | 31 | 26.3 | 27.1 |
| Turkey | 1 | 22 | 15 | 16 | 72.7 | 68.2 |
| Greece | 2 | 13 | 5 | 1 | 7.7 | 38.5 |
| Iran | 1 | 12 | 11 | 4 | 33.3 | 91.7 |
| Tunisia | 1 | 12 | 9 | 0 | 0.0 | 75.0 |
| Germany | 1 | 10 | 2 | 0 | 0.0 | 20.0 |
| Republic of Korea | 1 | 9 | 4 | 4 | 44.4 | 44.4 |
| UK | 1 | 5 | 2 | 2 | 40.0 | 40.0 |
| India | 1 | 4 | 3 | 3 | 75.0 | 75.0 |
| Libya | 1 | 2 | 2 | 2 | 100.0 | 100.0 |
| China | 1 | 1 | 1 | 0 | 0.0 | 100.0 |
| Saudi Arabia | 1 | 1 | 0 | 0 | 0.0 | 0.0 |

**Table C. Hydrocortisone-treated patients according to fludrocortisone-centre status**

| **Characteristic** | **Hydrocortisone patients in fludrocortisone-using centres** | **Hydrocortisone patients in non-fludrocortisone centres** | **p value** |
| --- | --- | --- | --- |
| Age, years | 65.0 ± 15.1 | 61.1 ± 16.2 | 0.019 |
| Male sex | 54/86 (62.8%) | 313/493 (63.5%) | 0.998 |
| Charlson comorbidity score | 3.2 ± 2.7 | 2.4 ± 2.7 | 0.001 |
| SAPS II at ICU admission | 52.9 ± 17.3 | 52.1 ± 17.3 | 0.696 |
| SOFA without cardiovascular component | 7.6 ± 2.8 | 7.6 ± 3.6 | 0.799 |
| Total SOFA score at BSI diagnosis | 10.5 ± 3.5 | 10.6 ± 4.2 | 0.949 |
| Maximum lactate at BSI diagnosis | 3.8 ± 3.1 | 4.1 ± 4.1 | 0.637 |
| Septic shock | 53/86 (61.6%) | 276/493 (56.0%) | 0.391 |
| Norepinephrine-equivalent dose ≥0.25 µg/kg/min | 31/86 (36.0%) | 100/493 (20.3%) | 0.002 |
| Respiratory source | 27/86 (31.4%) | 145/493 (29.4%) | 0.808 |
| Hydrocortisone duration, days | 5.0 [4.0–7.0] | 5.0 [3.0–7.0] | 0.101 |
| Day-28 mortality | 36/86 (41.9%) | 251/493 (50.9%) | 0.152 |

**Table D. Hydrocortisone duration and crude mortality**

| **Centre status** | **Hydrocortisone patients** | **Duration available** | **Duration mean** | **Duration SD** | **Duration median** | **Duration Q1** | **Duration Q3** | **Deaths** | **Mortality (%)** |
| --- | --- | --- | --- | --- | --- | --- | --- | --- | --- |
| Non-fludrocortisone centres | 493 | 493 | 5.5 | 4.3 | 5.0 | 3.0 | 7.0 | 251 | 50.9 |
| Fludrocortisone-using centres | 86 | 86 | 6.1 | 4.6 | 5.0 | 4.0 | 7.0 | 36 | 41.9 |

*Values are descriptive. P-values compare hydrocortisone-treated patients managed in fludrocortisone-using centres with those managed in non-fludrocortisone centres, using Wilcoxon rank-sum tests for continuous variables and chi-square/Fisher exact tests for categorical variables. The database did not include exact patient-level timing of hydrocortisone initiation.*
