# Supplementary material for: Association between hydrocortisone use and outcomes in sepsis patients with hospital-acquired bloodstream infections: An ancillary analysis of the EUROBACT-2 cohort
Source: PLoS One. 2026 Sep 15;21(9):e0356721. doi: 10.1371/journal.pone.0356721 (PMC13577328; doi:10.1371/journal.pone.0356721)
Supplement: Supplementary file 7 [file pone.0356721.s007.docx]

**Supplementary Table7 . Characteristics of matched and unmatched patients after propensity score matching**

Population: patients with complete data for the final propensity score model. The final propensity score included age, sex, Charlson comorbidity score, diagnosis at ICU admission, SOFA score without cardiovascular component, septic shock, country of inclusion, lactate at BSI diagnosis, and ICU-acquired versus non-ICU-acquired BSI. Matching used 1:1 nearest-neighbour matching without replacement with a caliper of 0.2 SD of the logit propensity score. Absolute standardized mean differences (SMD) and p-values are reported.

**Section A. Overall PS-complete cohort: Matched patients (N=1042) vs Unmatched patients (N=1170)**

| **Characteristic** | **Matched patients** | **Unmatched patients** | **\|SMD\|** | **p value** |
| --- | --- | --- | --- | --- |
| **Demographics and comorbidities** |  |  |  |  |
| Age, years | 62.8 ± 15.9 | 60.6 ± 16.4 | 0.135 | 0.002 |
| **Sex** |  |  | 0.008 | 0.895 |
| female | 368/1042 (35.3%) | 409/1170 (35.0%) | 0.008 |  |
| male | 674/1042 (64.7%) | 761/1170 (65.0%) | 0.008 |  |
| Body mass index | 27.3 ± 6.4 | 27.5 ± 7.4 | 0.042 | 0.238 |
| Charlson comorbidity score | 2.5 ± 2.7 | 2.1 ± 2.5 | 0.164 | <0.001 |
| **ICU admission and BSI context** |  |  |  |  |
| **Diagnosis at ICU admission** |  |  | 0.319 | <0.001 |
| COVID | 159/1042 (15.3%) | 142/1170 (12.1%) | 0.091 |  |
| Cardiac arrest | 32/1042 (3.1%) | 49/1170 (4.2%) | 0.060 |  |
| Cardio-vascular causes | 47/1042 (4.5%) | 73/1170 (6.2%) | 0.077 |  |
| Gastro-intestinal causes | 29/1042 (2.8%) | 47/1170 (4.0%) | 0.068 |  |
| Hypovolemic or Haemorragic shock | 28/1042 (2.7%) | 13/1170 (1.1%) | 0.115 |  |
| Metabolic causes | 9/1042 (0.9%) | 27/1170 (2.3%) | 0.116 |  |
| Multiple trauma (no TBI) | 42/1042 (4.0%) | 39/1170 (3.3%) | 0.037 |  |
| Neurologic causes | 54/1042 (5.2%) | 166/1170 (14.2%) | 0.304 |  |
| Other | 2/1042 (0.2%) | 1/1170 (0.1%) | 0.029 |  |
| Post-Operative admission | 93/1042 (8.9%) | 130/1170 (11.1%) | 0.073 |  |
| Renal failure | 11/1042 (1.1%) | 29/1170 (2.5%) | 0.108 |  |
| Respiratory admission | 220/1042 (21.1%) | 233/1170 (19.9%) | 0.030 |  |
| Sepsis or septic shock | 287/1042 (27.5%) | 170/1170 (14.5%) | 0.319 |  |
| Traumatic brain injury | 29/1042 (2.8%) | 51/1170 (4.4%) | 0.085 |  |
| **Timing/location of BSI acquisition** |  |  |  |  |
| **Country income category** |  |  | 0.216 | <0.001 |
| High-income | 613/1042 (58.8%) | 686/1170 (58.6%) | 0.004 |  |
| Low-income | 7/1042 (0.7%) | 0/1170 (0.0%) | 0.116 |  |
| Lower-middle-income | 116/1042 (11.1%) | 61/1170 (5.2%) | 0.216 |  |
| Upper-middle-income | 306/1042 (29.4%) | 423/1170 (36.2%) | 0.145 |  |
| **World Bank region** |  |  | 0.308 | <0.001 |
| East Asia and Pacific | 197/1042 (18.9%) | 158/1170 (13.5%) | 0.147 |  |
| Europe and Central Asia | 644/1042 (61.8%) | 890/1170 (76.1%) | 0.308 |  |
| Latin America and the Caribbean | 20/1042 (1.9%) | 28/1170 (2.4%) | 0.033 |  |
| Middle East and North Africa | 124/1042 (11.9%) | 73/1170 (6.2%) | 0.197 |  |
| North America | 10/1042 (1.0%) | 6/1170 (0.5%) | 0.052 |  |
| South Asia | 39/1042 (3.7%) | 14/1170 (1.2%) | 0.164 |  |
| Sub-Saharan Africa | 8/1042 (0.8%) | 1/1170 (0.1%) | 0.105 |  |
| Country of inclusion | Not shown in compact table | Not shown in compact table | 0.264 | <0.001 |
| **Severity at BSI diagnosis** |  |  |  |  |
| SAPS II at ICU admission | 51.8 ± 17.6 | 45.8 ± 15.6 | 0.362 | <0.001 |
| SOFA without cardiovascular component | 7.3 ± 3.4 | 5.5 ± 3.1 | 0.575 | <0.001 |
| Total SOFA score | 10.2 ± 4.1 | 7.1 ± 3.9 | 0.793 | <0.001 |
| Maximum lactate at BSI diagnosis | 3.9 ± 3.9 | 2.1 ± 1.8 | 0.593 | <0.001 |
| Septic shock | 598/1042 (57.4%) | 159/1170 (13.6%) | 0.915 | <0.001 |
| Norepinephrine-equivalent dose | 0.2 ± 0.8 | 0.1 ± 0.4 | 0.236 | <0.001 |
| Norepinephrine-equivalent dose ≥0.25 µg/kg/min | 219/1042 (21.0%) | 93/1170 (7.9%) | 0.371 | <0.001 |
| **Organ support at BSI diagnosis** |  |  |  |  |
| Invasive mechanical ventilation | 788/1042 (75.6%) | 748/1170 (63.9%) | 0.255 | <0.001 |
| Renal replacement therapy | 264/1042 (25.3%) | 179/1170 (15.3%) | 0.249 | <0.001 |
| ECMO | 27/1042 (2.6%) | 13/1170 (1.1%) | 0.110 | 0.014 |
| **Infection and management** |  |  |  |  |
| Respiratory source | 278/1042 (26.7%) | 295/1170 (25.2%) | 0.033 | 0.461 |
| **Full source classification** |  |  | 0.163 | 0.019 |
| CNS | 8/1042 (0.8%) | 9/1170 (0.8%) | 0.000 |  |
| Catheter-related | 4/1042 (0.4%) | 1/1170 (0.1%) | 0.062 |  |
| Endocarditis | 8/1042 (0.8%) | 8/1170 (0.7%) | 0.010 |  |
| Intra-abdominal | 181/1042 (17.4%) | 162/1170 (13.8%) | 0.097 |  |
| Mediastinitis | 5/1042 (0.5%) | 6/1170 (0.5%) | 0.005 |  |
| Other | 2/1042 (0.2%) | 4/1170 (0.3%) | 0.029 |  |
| Primary | 178/1042 (17.1%) | 182/1170 (15.6%) | 0.041 |  |
| Respiratory | 278/1042 (26.7%) | 295/1170 (25.2%) | 0.033 |  |
| Urinary tract | 71/1042 (6.8%) | 90/1170 (7.7%) | 0.034 |  |
| bone or soft tissues | 65/1042 (6.2%) | 57/1170 (4.9%) | 0.060 |  |
| catheter-related | 242/1042 (23.2%) | 356/1170 (30.4%) | 0.163 |  |
| **Time to adequate antimicrobial therapy** |  |  | 0.122 | <0.001 |
| 0–24 h | 106/983 (10.8%) | 151/1077 (14.0%) | 0.098 |  |
| 24–48 h | 126/983 (12.8%) | 177/1077 (16.4%) | 0.102 |  |
| 48–120 h | 36/983 (3.7%) | 61/1077 (5.7%) | 0.095 |  |
| Never | 535/983 (54.4%) | 539/1077 (50.0%) | 0.088 |  |
| ≥120 h | 180/983 (18.3%) | 149/1077 (13.8%) | 0.122 |  |
| **Source control** |  |  | 0.135 | 0.001 |
| Not required | 507/1042 (48.7%) | 529/1170 (45.2%) | 0.069 |  |
| Required and achieved | 421/1042 (40.4%) | 551/1170 (47.1%) | 0.135 |  |
| Required, not achieved | 114/1042 (10.9%) | 90/1170 (7.7%) | 0.112 |  |
| Clinical pharmacist never consulted | 413/1038 (39.8%) | 478/1161 (41.2%) | 0.028 | 0.538 |
| Centre use of fludrocortisone | 110/989 (11.1%) | 85/1004 (8.5%) | 0.089 | 0.055 |
| **Microbiology** |  |  |  |  |
| Gram-negative pathogen | 677/1042 (65.0%) | 696/1170 (59.5%) | 0.113 | 0.009 |
| Gram-positive pathogen | 318/1042 (30.5%) | 413/1170 (35.3%) | 0.102 | 0.019 |
| Fungal pathogen | 102/1042 (9.8%) | 95/1170 (8.1%) | 0.058 | 0.193 |
| Polymicrobial infection | 124/1042 (11.9%) | 123/1170 (10.5%) | 0.044 | 0.334 |
| Any antimicrobial resistance | 265/1042 (25.4%) | 286/1170 (24.4%) | 0.023 | 0.626 |
| Difficult-to-treat resistance | 169/643 (26.3%) | 134/658 (20.4%) | 0.140 | 0.014 |
| **Exposure and outcome** |  |  |  |  |
| Hydrocortisone use | 521/1042 (50.0%) | 40/1170 (3.4%) | 1.053 | <0.001 |
| Day-28 mortality | 480/1042 (46.1%) | 320/1170 (27.4%) | 0.388 | <0.001 |

**Section B. Hydrocortisone-treated patients only: Matched hydrocortisone (N=521) vs Unmatched hydrocortisone (N=40)**

| **Characteristic** | **Matched hydrocortisone** | **Unmatched hydrocortisone** | **\|SMD\|** | **p value** |
| --- | --- | --- | --- | --- |
| **Demographics and comorbidities** |  |  |  |  |
| Age, years | 62.5 ± 15.9 | 53.3 ± 16.6 | 0.565 | <0.001 |
| **Sex** |  |  | 0.210 | 0.256 |
| female | 181/521 (34.7%) | 18/40 (45.0%) | 0.210 |  |
| male | 340/521 (65.3%) | 22/40 (55.0%) | 0.210 |  |
| Body mass index | 27.5 ± 6.6 | 27.8 ± 5.6 | 0.048 | 0.719 |
| Charlson comorbidity score | 2.5 ± 2.7 | 2.5 ± 2.0 | 0.007 | 0.388 |
| **ICU admission and BSI context** |  |  |  |  |
| **Diagnosis at ICU admission** |  |  | 0.318 | 0.102 |
| COVID | 79/521 (15.2%) | 9/40 (22.5%) | 0.188 |  |
| Cardiac arrest | 17/521 (3.3%) | 0/40 (0.0%) | 0.258 |  |
| Cardio-vascular causes | 25/521 (4.8%) | 2/40 (5.0%) | 0.009 |  |
| Gastro-intestinal causes | 13/521 (2.5%) | 1/40 (2.5%) | 0.000 |  |
| Hypovolemic or Haemorragic shock | 13/521 (2.5%) | 0/40 (0.0%) | 0.225 |  |
| Metabolic causes | 6/521 (1.2%) | 0/40 (0.0%) | 0.152 |  |
| Multiple trauma (no TBI) | 20/521 (3.8%) | 0/40 (0.0%) | 0.280 |  |
| Neurologic causes | 24/521 (4.6%) | 0/40 (0.0%) | 0.307 |  |
| Other | 1/521 (0.2%) | 1/40 (2.5%) | 0.200 |  |
| Post-Operative admission | 44/521 (8.4%) | 1/40 (2.5%) | 0.261 |  |
| Renal failure | 5/521 (1.0%) | 0/40 (0.0%) | 0.139 |  |
| Respiratory admission | 108/521 (20.7%) | 14/40 (35.0%) | 0.318 |  |
| Sepsis or septic shock | 150/521 (28.8%) | 12/40 (30.0%) | 0.027 |  |
| Traumatic brain injury | 16/521 (3.1%) | 0/40 (0.0%) | 0.250 |  |
| **Timing/location of BSI acquisition** |  |  |  |  |
| **Country income category** |  |  | 0.651 | <0.001 |
| High-income | 304/521 (58.3%) | 11/40 (27.5%) | 0.623 |  |
| Low-income | 4/521 (0.8%) | 0/40 (0.0%) | 0.124 |  |
| Lower-middle-income | 60/521 (11.5%) | 16/40 (40.0%) | 0.651 |  |
| Upper-middle-income | 153/521 (29.4%) | 13/40 (32.5%) | 0.068 |  |
| **World Bank region** |  |  | 0.892 | <0.001 |
| East Asia and Pacific | 97/521 (18.6%) | 6/40 (15.0%) | 0.097 |  |
| Europe and Central Asia | 318/521 (61.0%) | 7/40 (17.5%) | 0.892 |  |
| Latin America and the Caribbean | 13/521 (2.5%) | 3/40 (7.5%) | 0.230 |  |
| Middle East and North Africa | 65/521 (12.5%) | 18/40 (45.0%) | 0.719 |  |
| North America | 4/521 (0.8%) | 0/40 (0.0%) | 0.124 |  |
| South Asia | 19/521 (3.6%) | 6/40 (15.0%) | 0.390 |  |
| Sub-Saharan Africa | 5/521 (1.0%) | 0/40 (0.0%) | 0.139 |  |
| Country of inclusion | Not shown in compact table | Not shown in compact table | 0.639 | <0.001 |
| **Severity at BSI diagnosis** |  |  |  |  |
| SAPS II at ICU admission | 52.5 ± 17.7 | 51.1 ± 16.8 | 0.083 | 0.482 |
| SOFA without cardiovascular component | 7.4 ± 3.4 | 9.6 ± 3.4 | 0.625 | <0.001 |
| Total SOFA score | 10.5 ± 4.1 | 13.2 ± 3.9 | 0.685 | <0.001 |
| Maximum lactate at BSI diagnosis | 4.0 ± 4.0 | 5.1 ± 4.1 | 0.253 | <0.001 |
| Septic shock | 300/521 (57.6%) | 36/40 (90.0%) | 0.737 | <0.001 |
| Norepinephrine-equivalent dose | 0.3 ± 0.9 | 0.2 ± 0.3 | 0.060 | 0.088 |
| Norepinephrine-equivalent dose ≥0.25 µg/kg/min | 119/521 (22.8%) | 12/40 (30.0%) | 0.162 | 0.402 |
| **Organ support at BSI diagnosis** |  |  |  |  |
| Invasive mechanical ventilation | 415/521 (79.7%) | 29/40 (72.5%) | 0.168 | 0.006 |
| Renal replacement therapy | 140/521 (26.9%) | 12/40 (30.0%) | 0.069 | 0.807 |
| ECMO | 15/521 (2.9%) | 3/40 (7.5%) | 0.208 | 0.130 |
| **Infection and management** |  |  |  |  |
| Respiratory source | 152/521 (29.2%) | 18/40 (45.0%) | 0.328 | 0.055 |
| **Full source classification** |  |  | 0.328 | 0.809 |
| CNS | 2/521 (0.4%) | 0/40 (0.0%) | 0.088 |  |
| Catheter-related | 3/521 (0.6%) | 0/40 (0.0%) | 0.107 |  |
| Endocarditis | 5/521 (1.0%) | 0/40 (0.0%) | 0.139 |  |
| Intra-abdominal | 89/521 (17.1%) | 7/40 (17.5%) | 0.011 |  |
| Mediastinitis | 2/521 (0.4%) | 0/40 (0.0%) | 0.088 |  |
| Other | 1/521 (0.2%) | 0/40 (0.0%) | 0.062 |  |
| Primary | 80/521 (15.4%) | 5/40 (12.5%) | 0.082 |  |
| Respiratory | 152/521 (29.2%) | 18/40 (45.0%) | 0.328 |  |
| Urinary tract | 40/521 (7.7%) | 1/40 (2.5%) | 0.236 |  |
| bone or soft tissues | 33/521 (6.3%) | 2/40 (5.0%) | 0.058 |  |
| catheter-related | 114/521 (21.9%) | 7/40 (17.5%) | 0.110 |  |
| **Time to adequate antimicrobial therapy** |  |  | 0.227 | 0.424 |
| 0–24 h | 57/502 (11.4%) | 3/38 (7.9%) | 0.117 |  |
| 24–48 h | 61/502 (12.2%) | 5/38 (13.2%) | 0.030 |  |
| 48–120 h | 14/502 (2.8%) | 3/38 (7.9%) | 0.227 |  |
| Never | 283/502 (56.4%) | 19/38 (50.0%) | 0.128 |  |
| ≥120 h | 87/502 (17.3%) | 8/38 (21.1%) | 0.095 |  |
| **Source control** |  |  | 0.237 | 0.359 |
| Not required | 243/521 (46.6%) | 14/40 (35.0%) | 0.237 |  |
| Required and achieved | 217/521 (41.7%) | 20/40 (50.0%) | 0.168 |  |
| Required, not achieved | 61/521 (11.7%) | 6/40 (15.0%) | 0.097 |  |
| Clinical pharmacist never consulted | 198/520 (38.1%) | 10/40 (25.0%) | 0.281 | 0.139 |
| Centre use of fludrocortisone | 72/504 (14.3%) | 9/39 (23.1%) | 0.226 | 0.211 |
| **Microbiology** |  |  |  |  |
| Gram-negative pathogen | 349/521 (67.0%) | 27/40 (67.5%) | 0.011 | 1.000 |
| Gram-positive pathogen | 148/521 (28.4%) | 10/40 (25.0%) | 0.077 | 0.780 |
| Fungal pathogen | 52/521 (10.0%) | 3/40 (7.5%) | 0.088 | 0.786 |
| Polymicrobial infection | 61/521 (11.7%) | 1/40 (2.5%) | 0.358 | 0.110 |
| Any antimicrobial resistance | 136/521 (26.1%) | 15/40 (37.5%) | 0.245 | 0.167 |
| Difficult-to-treat resistance | 93/334 (27.8%) | 8/26 (30.8%) | 0.064 | 0.926 |
| **Exposure and outcome** |  |  |  |  |
| Hydrocortisone use | 521/521 (100.0%) | 40/40 (100.0%) |  |  |
| Day-28 mortality | 260/521 (49.9%) | 20/40 (50.0%) | 0.002 | 1.000 |

**Section C. Control patients only: Matched controls (N=521) vs Unmatched controls (N=1130)**

| **Characteristic** | **Matched controls** | **Unmatched controls** | **\|SMD\|** | **p value** |
| --- | --- | --- | --- | --- |
| **Demographics and comorbidities** |  |  |  |  |
| Age, years | 63.2 ± 15.9 | 60.9 ± 16.3 | 0.141 | 0.008 |
| **Sex** |  |  | 0.027 | 0.649 |
| female | 187/521 (35.9%) | 391/1130 (34.6%) | 0.027 |  |
| male | 334/521 (64.1%) | 739/1130 (65.4%) | 0.027 |  |
| Body mass index | 27.0 ± 6.1 | 27.5 ± 7.4 | 0.074 | 0.133 |
| Charlson comorbidity score | 2.5 ± 2.7 | 2.1 ± 2.5 | 0.177 | <0.001 |
| **ICU admission and BSI context** |  |  |  |  |
| **Diagnosis at ICU admission** |  |  | 0.307 | <0.001 |
| COVID | 80/521 (15.4%) | 133/1130 (11.8%) | 0.105 |  |
| Cardiac arrest | 15/521 (2.9%) | 49/1130 (4.3%) | 0.078 |  |
| Cardio-vascular causes | 22/521 (4.2%) | 71/1130 (6.3%) | 0.092 |  |
| Gastro-intestinal causes | 16/521 (3.1%) | 46/1130 (4.1%) | 0.054 |  |
| Hypovolemic or Haemorragic shock | 15/521 (2.9%) | 13/1130 (1.2%) | 0.123 |  |
| Metabolic causes | 3/521 (0.6%) | 27/1130 (2.4%) | 0.150 |  |
| Multiple trauma (no TBI) | 22/521 (4.2%) | 39/1130 (3.5%) | 0.040 |  |
| Neurologic causes | 30/521 (5.8%) | 166/1130 (14.7%) | 0.295 |  |
| Other | 1/521 (0.2%) | 0/1130 (0.0%) | 0.062 |  |
| Post-Operative admission | 49/521 (9.4%) | 129/1130 (11.4%) | 0.066 |  |
| Renal failure | 6/521 (1.2%) | 29/1130 (2.6%) | 0.105 |  |
| Respiratory admission | 112/521 (21.5%) | 219/1130 (19.4%) | 0.052 |  |
| Sepsis or septic shock | 137/521 (26.3%) | 158/1130 (14.0%) | 0.307 |  |
| Traumatic brain injury | 13/521 (2.5%) | 51/1130 (4.5%) | 0.110 |  |
| **Timing/location of BSI acquisition** |  |  |  |  |
| **Country income category** |  |  | 0.259 | <0.001 |
| High-income | 309/521 (59.3%) | 675/1130 (59.7%) | 0.009 |  |
| Low-income | 3/521 (0.6%) | 0/1130 (0.0%) | 0.107 |  |
| Lower-middle-income | 56/521 (10.7%) | 45/1130 (4.0%) | 0.259 |  |
| Upper-middle-income | 153/521 (29.4%) | 410/1130 (36.3%) | 0.147 |  |
| **World Bank region** |  |  | 0.341 | <0.001 |
| East Asia and Pacific | 100/521 (19.2%) | 152/1130 (13.5%) | 0.155 |  |
| Europe and Central Asia | 326/521 (62.6%) | 883/1130 (78.1%) | 0.341 |  |
| Latin America and the Caribbean | 7/521 (1.3%) | 25/1130 (2.2%) | 0.066 |  |
| Middle East and North Africa | 59/521 (11.3%) | 55/1130 (4.9%) | 0.237 |  |
| North America | 6/521 (1.2%) | 6/1130 (0.5%) | 0.068 |  |
| South Asia | 20/521 (3.8%) | 8/1130 (0.7%) | 0.210 |  |
| Sub-Saharan Africa | 3/521 (0.6%) | 1/1130 (0.1%) | 0.085 |  |
| Country of inclusion | Not shown in compact table | Not shown in compact table | 0.268 | <0.001 |
| **Severity at BSI diagnosis** |  |  |  |  |
| SAPS II at ICU admission | 51.2 ± 17.5 | 45.6 ± 15.5 | 0.333 | <0.001 |
| SOFA without cardiovascular component | 7.2 ± 3.4 | 5.3 ± 3.0 | 0.595 | <0.001 |
| Total SOFA score | 10.0 ± 4.1 | 6.8 ± 3.7 | 0.800 | <0.001 |
| Maximum lactate at BSI diagnosis | 3.8 ± 3.8 | 2.0 ± 1.6 | 0.607 | <0.001 |
| Septic shock | 298/521 (57.2%) | 123/1130 (10.9%) | 0.977 | <0.001 |
| Norepinephrine-equivalent dose | 0.2 ± 0.8 | 0.1 ± 0.4 | 0.191 | <0.001 |
| Norepinephrine-equivalent dose ≥0.25 µg/kg/min | 100/521 (19.2%) | 81/1130 (7.2%) | 0.355 | <0.001 |
| **Organ support at BSI diagnosis** |  |  |  |  |
| Invasive mechanical ventilation | 373/521 (71.6%) | 719/1130 (63.6%) | 0.170 | 0.014 |
| Renal replacement therapy | 124/521 (23.8%) | 167/1130 (14.8%) | 0.229 | <0.001 |
| ECMO | 12/521 (2.3%) | 10/1130 (0.9%) | 0.113 | 0.035 |
| **Infection and management** |  |  |  |  |
| Respiratory source | 126/521 (24.2%) | 277/1130 (24.5%) | 0.008 | 0.934 |
| **Full source classification** |  |  | 0.141 | 0.135 |
| CNS | 6/521 (1.2%) | 9/1130 (0.8%) | 0.036 |  |
| Catheter-related | 1/521 (0.2%) | 1/1130 (0.1%) | 0.028 |  |
| Endocarditis | 3/521 (0.6%) | 8/1130 (0.7%) | 0.017 |  |
| Intra-abdominal | 92/521 (17.7%) | 155/1130 (13.7%) | 0.108 |  |
| Mediastinitis | 3/521 (0.6%) | 6/1130 (0.5%) | 0.006 |  |
| Other | 1/521 (0.2%) | 4/1130 (0.4%) | 0.031 |  |
| Primary | 98/521 (18.8%) | 177/1130 (15.7%) | 0.083 |  |
| Respiratory | 126/521 (24.2%) | 277/1130 (24.5%) | 0.008 |  |
| Urinary tract | 31/521 (6.0%) | 89/1130 (7.9%) | 0.076 |  |
| bone or soft tissues | 32/521 (6.1%) | 55/1130 (4.9%) | 0.056 |  |
| catheter-related | 128/521 (24.6%) | 349/1130 (30.9%) | 0.141 |  |
| **Time to adequate antimicrobial therapy** |  |  | 0.155 | 0.007 |
| 0–24 h | 49/481 (10.2%) | 148/1039 (14.2%) | 0.124 |  |
| 24–48 h | 65/481 (13.5%) | 172/1039 (16.6%) | 0.085 |  |
| 48–120 h | 22/481 (4.6%) | 58/1039 (5.6%) | 0.046 |  |
| Never | 252/481 (52.4%) | 520/1039 (50.0%) | 0.047 |  |
| ≥120 h | 93/481 (19.3%) | 141/1039 (13.6%) | 0.155 |  |
| **Source control** |  |  | 0.158 | 0.006 |
| Not required | 264/521 (50.7%) | 515/1130 (45.6%) | 0.102 |  |
| Required and achieved | 204/521 (39.2%) | 531/1130 (47.0%) | 0.158 |  |
| Required, not achieved | 53/521 (10.2%) | 84/1130 (7.4%) | 0.097 |  |
| Clinical pharmacist never consulted | 215/518 (41.5%) | 468/1121 (41.7%) | 0.005 | 0.969 |
| Centre use of fludrocortisone | 38/485 (7.8%) | 76/965 (7.9%) | 0.002 | 1.000 |
| **Microbiology** |  |  |  |  |
| Gram-negative pathogen | 328/521 (63.0%) | 669/1130 (59.2%) | 0.077 | 0.163 |
| Gram-positive pathogen | 170/521 (32.6%) | 403/1130 (35.7%) | 0.064 | 0.251 |
| Fungal pathogen | 50/521 (9.6%) | 92/1130 (8.1%) | 0.051 | 0.376 |
| Polymicrobial infection | 63/521 (12.1%) | 122/1130 (10.8%) | 0.041 | 0.489 |
| Any antimicrobial resistance | 129/521 (24.8%) | 271/1130 (24.0%) | 0.018 | 0.779 |
| Difficult-to-treat resistance | 76/309 (24.6%) | 126/632 (19.9%) | 0.112 | 0.121 |
| **Exposure and outcome** |  |  |  |  |
| Hydrocortisone use | 0/521 (0.0%) | 0/1130 (0.0%) |  |  |
| Day-28 mortality | 220/521 (42.2%) | 300/1130 (26.5%) | 0.330 | <0.001 |

*SMD = standardized mean difference. For categorical variables, the variable-level SMD is the maximum absolute SMD across categories. P-values were calculated using the Wilcoxon rank-sum test for continuous variables and chi-square or Fisher exact tests for categorical variables. Country levels are not expanded in the compact DOCX table; full country-by-country and category-level distributions are provided in the detailed CSV file.*
