# Supplementary material for: Association between hydrocortisone use and outcomes in sepsis patients with hospital-acquired bloodstream infections: An ancillary analysis of the EUROBACT-2 cohort
Source: PLoS One. 2026 Sep 15;21(9):e0356721. doi: 10.1371/journal.pone.0356721 (PMC13577328; doi:10.1371/journal.pone.0356721)
Supplement: Supplementary file 8 [file pone.0356721.s008.docx]

**Supplementary Table 8. Multivariable analysis in the high-dose norepinephrine subgroup**

Subgroup defined as norepinephrine-equivalent dose ≥0.25 µg/kg/min. The matched subgroup included 219 patients; 199 patients were included in the complete-case multivariable model. The norepinephrine variable was not included as a covariate because it defines the subgroup.

| **Characteristic** | **OR** | **95% CI** | **p** |
| --- | --- | --- | --- |
| Hydrocortisone exposure | 1.083 | 0.567 ; 2.067 | 0.810 |
| Respiratory first source | 1.759 | 0.836 ; 3.701 | 0.137 |
| **Time to adequate antimicrobial therapy** |  |  |  |
| Never (reference) | — | — | — |
| 0–24 h | 1.205 | 0.372 ; 3.904 | 0.756 |
| 24–48 h | 1.744 | 0.549 ; 5.538 | 0.346 |
| 48–120 h | 2.828 | 0.454 ; 17.607 | 0.265 |
| ≥120 h | 1.360 | 0.572 ; 3.234 | 0.486 |
| **Source control** |  |  |  |
| Not needed (reference) | — | — | — |
| Not controlled | 15.315 | 1.779 ; 131.835 | 0.013 |
| Controlled | 0.405 | 0.206 ; 0.794 | 0.009 |
| Clinical pharmacist never consulted | 1.201 | 0.634 ; 2.276 | 0.574 |
| Centre use of fludrocortisone | 0.643 | 0.281 ; 1.473 | 0.297 |

*OR = odds ratio; CI = confidence interval. For time to adequate antimicrobial therapy, the reference category is 'never'. For source control, the reference category is 'not needed'. Estimates were obtained from logistic regression with cluster-robust standard errors by matched pair. The norepinephrine-equivalent dose ≥0.25 µg/kg/min variable was omitted from the model because it defined the subgroup.*
