# Supplementary material for: Association between hydrocortisone use and outcomes in sepsis patients with hospital-acquired bloodstream infections: An ancillary analysis of the EUROBACT-2 cohort
Source: PLoS One. 2026 Sep 15;21(9):e0356721. doi: 10.1371/journal.pone.0356721 (PMC13577328; doi:10.1371/journal.pone.0356721)
Supplement: Supplementary file 10 [file pone.0356721.s015.docx]

Detailed Response to Reviewers

*EUROBACT/hydrocortisone manuscript — expanded point-by-point response*

Dear Academic Editor and Reviewers,

We sincerely thank the Academic Editor and the reviewers for their careful reading of our manuscript and for the constructive comments that helped us improve both the methodological transparency and the interpretation of our work. We have substantially revised the statistical analysis, the presentation of the results, and the wording of the manuscript. In particular, we strengthened the propensity score model, clarified the matching strategy, added balance diagnostics, removed post-exposure hydrocortisone duration from the main model, performed several sensitivity analyses, and toned down causal language throughout the manuscript. Below, we provide a point-by-point response and summarize the concrete actions performed for each issue raised.

# Journal requirements

**Comment 1.** *Formatting, figure/table citations, and supporting information.*

Response. Thank you for outlining this requirement. We revised the manuscript formatting according to PLOS ONE requirements. Main tables are now intended to be included in the manuscript file; supplementary tables and figures have been prepared as supporting information with explicit captions. We also checked that all figures and tables are cited in the text, including Figure 1 and Table 3. The revised submission includes a clean manuscript, a marked-up manuscript, and a separate response-to-reviewers file.

**Comment 2.** *Funding and competing interests.*

Response. We thank the journal office for this clarification. We have provided updated Funding and Competing Interests statements in the cover letter and online submission form. The updated statements clarify that the declared relationships did not influence study design, data collection, data analysis, interpretation, decision to publish, or manuscript preparation. We have also included the required PLOS ONE statement that these declarations do not alter our adherence to PLOS ONE policies on sharing data and materials.

**Comment 3.** *EUROBACT-2 Study Group.*

Response. Thank you for this administrative point. We have updated the acknowledgments to list EUROBACT-2 Study Group contributors and affiliations and to identify a lead contact for the group, in accordance with PLOS ONE requirements.

**Comment 4.** *Data availability.*

Response. We have addressed this requirement by revising the Data Availability Statement. Because the dataset contains sensitive individual-level clinical data collected across multiple countries and institutions, unrestricted public sharing is not permitted. The revised statement provides the appropriate data-access contact and specify conditions under which qualified researchers may request access.

**Comment 5.** *Ethics statement.*

Response. We appreciate this clarification. The previous submission form entry of 'N/A' was inconsistent with the manuscript. The revised submission states that EUROBACT-2 was approved as a low-risk research project with waiver of individual consent by the Human Research Ethics Committee of the Royal Brisbane & Women’s Hospital, Queensland, Australia (LNR/2019/QRBW/48376), with local approvals obtained according to national and local regulations.

# Response to the Academic Editor

**Comment 1.** *Important variables influencing hydrocortisone administration may not have been fully captured.*

**Response.** We thank the Academic Editor for this important comment. We strengthened the propensity score model by adding age, lactate at HA-BSI diagnosis, and ICU-acquired versus non-ICU-acquired HA-BSI to the original variables. The final PS included age, sex, Charlson score, ICU admission diagnosis, SOFA without cardiovascular component, septic shock, country, lactate at HA-BSI diagnosis, and ICU-acquired/non-ICU-acquired HA-BSI. Matching used 1:1 nearest-neighbour matching without replacement and a caliper of 0.2 SD of the logit PS, resulting in 521 pairs and 1,042 matched patients. We also added love plots and explicitly acknowledge that clinician judgment, unmeasured hemodynamic instability and timing of shock onset may remain residual confounders.

**Comment 2.** *The main association was borderline and appeared fragile in the original analysis.*

**Response.** We are grateful for this careful assessment. We repeated the analysis using the revised PS and updated multivariable model. In the revised main analysis, hydrocortisone remained associated with day-28 mortality in the overall matched cohort. The association also persisted after excluding COVID-19 patients. Importantly, the association was not observed among patients with septic shock or among those receiving norepinephrine-equivalent dose ≥0.25 µg/kg/min. We revised the interpretation to state that the signal appears mainly outside the subgroup closest to guideline-based hydrocortisone indications.

**Comment 3.** *Hydrocortisone may be a marker of severity rather than a cause of harm.*

**Response.** Thank you for raising this important methodological point. We removed causal wording throughout the manuscript. The results are now described as associations. The Discussion explicitly addresses confounding by indication, residual confounding, and the possibility that hydrocortisone use may reflect perceived severity rather than causing harm.

**Comment 4.** *Hydrocortisone exposure is poorly characterized, especially timing of initiation.*

**Response.** We appreciate this comment, which helped us clarify a key limitation. The exact date or time of hydrocortisone initiation relative to blood culture sampling or HA-BSI diagnosis was not available in EUROBACT-2. We therefore cannot analyze the temporal relationship between exposure and infection diagnosis. This has been added to the Methods and Discussion, together with the statement that time-dependent bias and residual time-dependent confounding cannot be excluded.

**Comment 5.** *Hydrocortisone duration varies and may create heterogeneous exposure groups.*

Response. We thank the Academic Editor for highlighting this issue. In the initial version of the manuscript, duration of hydrocortisone exposure was included in the multivariable model. After reconsideration, and in response to the concern raised during the review process, we agree that this variable should not be included in the primary adjustment model, as it occurs after treatment initiation and may be influenced by subsequent clinical evolution and survival. We therefore removed hydrocortisone duration from the primary multivariable model. With this revised and methodologically more appropriate model, hydrocortisone use was associated with day-28 mortality in the overall matched cohort. This finding remains observational and should not be interpreted causally. Hydrocortisone duration is now considered descriptively rather than as a baseline confounder.

**Comment 6.** *Practice variability and centre effects may influence treatment decisions.*

**Response.** This is an important point. Country was included in the PS model, and we added a dedicated love plot for centre/country/income variables. Same-centre or same-country exact matching was not feasible without major sample loss, and this is acknowledged as a limitation. We now explicitly discuss residual centre- and country-level confounding.

**Comment 7.** *Please justify the matching strategy and robustness of findings.*

**Response.** We clarified the matching strategy in the Methods: 1:1 nearest-neighbour matching without replacement, caliper 0.2 SD of the logit PS. We added PS distribution plots and three love plots. We also performed several sensitivity analyses: excluding COVID-19 patients, excluding CoNS patients from the already matched cohort, removing time to adequate antimicrobial therapy and source control from the model, analyzing the high-dose norepinephrine subgroup, and exploring secondary outcomes.

**Comment 8.** *The conclusion should be cautious.*

**Response.** We thank the Academic Editor for this important comment. The conclusion has been revised to state that hydrocortisone use was associated with higher day-28 mortality in the overall matched cohort, but not among patients with septic shock or high-dose norepinephrine. We explicitly describe the findings as observational and hypothesis-generating.

# Response to Reviewer 1

**Comment 1.** *The introduction should highlight that HA-BSI is a high-risk population differing from typical sepsis RCT cohorts.*

**Response.** We thank Reviewer 1 for this helpful comment. We revised the Introduction to emphasize that HA-BSI is a high-risk, hospital-acquired and microbiologically documented infection context. We also clarified that this population differs from many randomized trials of corticosteroids in septic shock, which often included broader sepsis populations and variable microbiological documentation.

**Comment 2.** *Worldwide studies are affected by healthcare-system, country and centre effects.*

**Response.** Thank you for this important suggestion. We addressed this by including country in the PS model and by presenting centre/country/income balance in a dedicated love plot. We also expanded the Discussion to acknowledge residual country- and centre-level confounding.

**Comment 3.** *It would be useful to know whether matched pairs came from the same centre or country.*

**Response.** We appreciate the reviewer’s careful reading. We assessed this issue in the final matched cohort. Exact same-centre or same-country matching was not used because it would have produced major sample loss. The revised manuscript clarifies that country was addressed through the PS model and that residual centre/country effects remain possible.

**Comment 4.** *Patients receiving hydrocortisone appeared more severely ill even after matching.*

**Response.** We agree that this needed further clarification. We added lactate at HA-BSI diagnosis and ICU-acquired/non-ICU-acquired HA-BSI to the PS model. We also added love plots separating PS/multivariable variables, centre/country variables, and clinically relevant variables not included in the model, including organ support and severity markers.

**Comment 5.** *COVID pandemic and economic/regional differences should be mentioned.*

**Response.** This is a very relevant comment. We added this context and performed a sensitivity analysis excluding COVID-19 patients. In this analysis, the global association persisted, but no association was found in patients with septic shock or high-dose norepinephrine.

**Comment 6.** *Hydrocortisone administration differed across centres and duration differed from RCTs.*

**Response.** We thank the reviewer for this comment. We now describe real-life treatment heterogeneity more clearly. Hydrocortisone duration was removed from the main model because it is post-exposure and may reflect clinical evolution.

**Comment 7.** *Centres using fludrocortisone require further description, including country and duration.*

**Response.** We performed a descriptive analysis of fludrocortisone use. It was limited to a small number of centres and countries, mostly France, and involved relatively few hydrocortisone-treated patients. Crude mortality was numerically lower in fludrocortisone-using centres but not statistically significant, and hydrocortisone duration was broadly similar. We use this only to acknowledge treatment heterogeneity and do not draw conclusions on fludrocortisone efficacy.

**Comment 8.** *High-dose norepinephrine may reflect severity or limitation of therapy.*

**Response.** We performed a dedicated subgroup analysis among patients receiving norepinephrine-equivalent dose ≥0.25 µg/kg/min. Hydrocortisone was not associated with day-28 mortality in this subgroup. This is now highlighted because this subgroup is closest to current guideline-based corticosteroid indications.

**Comment 9.** *Discuss ADRENAL sex-related findings.*

**Response.** We added a cautious discussion of sex-related findings. However, the formal hydrocortisone × sex interaction test was not significant, so the female subgroup signal is presented as exploratory.

**Comment 10.** *Avoid dexamethasone studies when discussing hydrocortisone.*

**Response.** We revised the Discussion to focus on hydrocortisone and hydrocortisone/fludrocortisone evidence and avoided overinterpreting dexamethasone studies.

**Comment 11.** *Discuss late-onset sepsis and immunoparalysis.*

**Response.** We added a cautious discussion of late-onset/hospital-acquired sepsis and possible immune dysfunction in prolonged critical illness, without presenting this as a mechanism demonstrated in our data.

# Response to Reviewer 2

**Comment 1.** *Clarify source control handling, given its complex relation with severity and outcomes.*

**Response.** We thank Reviewer 2 for this important methodological comment. Source control was retained in the main model because it is prognostically important, but we now discuss its potentially post-baseline nature. We also performed a sensitivity analysis removing both source control and time to adequate antimicrobial therapy; the overall hydrocortisone association persisted, suggesting that the main result was not solely driven by these covariates.

**Comment 2.** *Clarify norepinephrine dose in the causal framework and model.*

**Response.** Thank you for this thoughtful remark. Norepinephrine-equivalent dose ≥0.25 µg/kg/min was included as a multivariable covariate, and a dedicated subgroup analysis was performed. Hydrocortisone was not associated with mortality in this high-dose norepinephrine subgroup.

**Comment 3.** *Hydrocortisone duration is post-exposure and should be reconsidered.*

Response. We thank the reviewer for this important methodological comment. In the initial version of the manuscript, duration of hydrocortisone exposure was included in the multivariable model. After reconsideration, and in response to the reviewer’s concern, we agree that this variable should not be included in the primary adjustment model, as it occurs after treatment initiation and may be influenced by subsequent clinical evolution and survival. We therefore removed hydrocortisone duration from the primary multivariable model. With this revised and methodologically more appropriate model, hydrocortisone use was associated with day-28 mortality in the overall matched cohort. This result remains observational and should not be interpreted causally.

**Comment 4.** *Provide characteristics of patients excluded by matching and discuss generalizability.*

**Response.** We added a supplementary matched-versus-unmatched analysis among patients complete for the final PS. This includes matched vs unmatched patients overall, matched vs unmatched hydrocortisone-treated patients, and matched vs unmatched controls. Both absolute SMDs and p-values are provided. This analysis shows that the matched results apply to the common-support population, not necessarily the entire EUROBACT-2 HA-BSI cohort.

**Comment 5.** *Clarify whether the caliper was applied to raw PS or logit PS.*

**Response.** We clarified that matching used a caliper of 0.2 SD of the logit propensity score.

**Comment 6.** *The SSC norepinephrine threshold should be described as a practice remark rather than a formal definition.*

**Response.** We revised the wording accordingly and now describe the norepinephrine-equivalent threshold as a clinically relevant guideline-based practice threshold rather than a formal definition.

**Comment 7.** *The 'never' reference category for adequate antimicrobial therapy may limit interpretability.*

**Response.** We clarified the categorization and reference category. We also performed a sensitivity analysis excluding time to adequate antimicrobial therapy together with source control; the global hydrocortisone association persisted.

**Comment 8.** *Perform sensitivity analysis excluding coagulase-negative staphylococci.*

**Response.** We performed this sensitivity analysis from the already matched cohort, without rematching. After excluding patients with CoNS infection, the global hydrocortisone association persisted, whereas no association was observed among patients with septic shock or high-dose norepinephrine. This suggests that the findings are not solely explained by inclusion of CoNS infections.

# Response to Reviewer 3

**Comment 1.** *Strengthen rationale for hydrocortisone in nosocomial sepsis and sepsis without shock.*

**Response.** We first would like to thank reviewer 3 for the time employed to review this work. We revised the Introduction to explain that HA-BSI is a distinct, microbiologically documented, hospital-acquired context. We also clarify that studying real-life hydrocortisone use with and without septic shock is relevant because hydrocortisone may be used outside strict guideline-based indications.

**Comment 2.** *Clarify inclusion criteria.*

**Response.** We clarified that all eligible adult ICU patients with HA-BSI were considered. Hydrocortisone administration defined the exposed group, and patients not receiving hydrocortisone served as controls.

**Comment 3.** *Clarify hydrocortisone exposure timing.*

**Response.** We clarified that exact timing of hydrocortisone initiation relative to blood culture sampling or HA-BSI diagnosis was not available. This is now included as a limitation.

**Comment 4.** *Distinguish ICU-acquired from non-ICU-acquired HA-BSI.*

**Response.** We included ICU-acquired versus non-ICU-acquired HA-BSI in the revised PS and clarified the timing of severity and organ-support variables.

**Comment 5.** *Specify chronic corticosteroid definition and exclusion of other steroids.*

**Response.** We clarified exclusion of chronic corticosteroid therapy and corticosteroids other than hydrocortisone.

**Comment 6.** *Confirm septic shock definition and lactate availability.*

**Response.** We clarified the Sepsis-3 septic shock definition and added lactate at HA-BSI diagnosis to the revised PS model.

**Comment 7.** *Perform subgroup analysis by vasopressor requirement.*

**Response.** We performed the norepinephrine-equivalent ≥0.25 µg/kg/min subgroup analysis. Hydrocortisone was not associated with day-28 mortality in this subgroup.

**Comment 8.** *Clarify PS variables and final model variables.*

**Response.** We now list PS variables and multivariable adjustment covariates separately in the Methods.

**Comment 9.** *Provide PS distribution and SMD before/after matching.*

**Response.** We added PS distribution plots and love plots showing standardized mean differences before and after matching.

**Comment 10.** *Justify female subgroup analysis.*

**Response.** The female subgroup finding is now presented as exploratory because the formal hydrocortisone × sex interaction was not significant.

**Comment 11.** *Address residual confounding due to organ support and vasopressor imbalance.*

**Response.** We added balance plots for organ support and severity markers and explicitly discuss residual confounding.

**Comment 12.** *Add secondary outcomes if available.*

**Response.** We added exploratory analyses of vasopressor-free days, mechanical ventilation-free days and SOFA variation from HA-BSI diagnosis to day 7. Hydrocortisone was associated with fewer support-free days and a less favorable SOFA trajectory. Exact timing of shock resolution and recurrence of nosocomial infection were not available.

**Comment 13.** *Strengthen discussion of confounding, time-dependent bias and phenotype heterogeneity.*

**Response.** We revised the Discussion to address confounding by indication, residual confounding despite matching, unavailable exposure timing, centre/country effects, and sepsis phenotype heterogeneity.

**Comment 14.** *Interpretation should not be causal.*

**Response.** We revised the manuscript to consistently present the results as observational associations and hypothesis-generating findings.

# Closing statement

We again thank the Academic Editor and the reviewers for their constructive comments. We believe that the revised manuscript is now more transparent regarding matching, balance assessment, exposure limitations, residual confounding, subgroup interpretation and generalizability. The conclusions have been substantially tempered and no causal claim is made regarding hydrocortisone use.
