# Supplementary material for: Association between hydrocortisone use and outcomes in sepsis patients with hospital-acquired bloodstream infections: An ancillary analysis of the EUROBACT-2 cohort
Source: PLoS One. 2026 Sep 15;21(9):e0356721. doi: 10.1371/journal.pone.0356721 (PMC13577328; doi:10.1371/journal.pone.0356721)
Supplement: Supplementary file 11 [file pone.0356721.s016.docx]

Detailed Response to Reviewers

*EUROBACT/hydrocortisone manuscript — expanded point-by-point response*

Dear Academic Editor and Reviewers,

Thank you for the time you have spent on this manuscript. Your reviews were useful to improve its quality.

To reviewer 2:

The names of the variables have been corrected in the figure 3 and 5. Thank you
