# Supplementary material for: Association between hydrocortisone use and outcomes in sepsis patients with hospital-acquired bloodstream infections: An ancillary analysis of the EUROBACT-2 cohort
Source: PLoS One. 2026 Sep 15;21(9):e0356721. doi: 10.1371/journal.pone.0356721 (PMC13577328; doi:10.1371/journal.pone.0356721)
Supplement: Peer review history

# Peer review: Decision Letter 0

**DOI:** 10.1371/journal.pone.0356721.r001

**Authors:** Eleni Magira

6 Apr 2026

Dear Dr. Duclos,

Thank you for submitting your manuscript to PLOS ONE. After careful consideration, we feel that it has merit but does not fully meet PLOS ONE’s publication criteria as it currently stands. Therefore, we invite you to submit a revised version of the manuscript that addresses the points raised during the review process.

Please submit your revised manuscript by May 21 2026 11:59PM. If you will need more time than this to complete your revisions, please reply to this message or contact the journal office at plosone@plos.org. When you're ready to submit your revision, log on to https://www.editorialmanager.com/pone/ and select the 'Submissions Needing Revision' folder to locate your manuscript file.

- A letter that responds to each point raised by the academic editor and reviewer(s). You should upload this letter as a separate file labeled 'Response to Reviewers'.
- A marked-up copy of your manuscript that highlights changes made to the original version. You should upload this as a separate file labeled 'Revised Manuscript with Track Changes'.
- An unmarked version of your revised paper without tracked changes. You should upload this as a separate file labeled 'Manuscript'.

If you would like to make changes to your financial disclosure, please include your updated statement in your cover letter. Guidelines for resubmitting your figure files are available below the reviewer comments at the end of this letter.

If applicable, we recommend that you deposit your laboratory protocols in protocols.io to enhance the reproducibility of your results. Protocols.io assigns your protocol its own identifier (DOI) so that it can be cited independently in the future. For instructions see: https://journals.plos.org/plosone/s/submission-guidelines#loc-laboratory-protocols. Additionally, PLOS ONE offers an option for publishing peer-reviewed Lab Protocol articles, which describe protocols hosted on protocols.io. Read more information on sharing protocols at https://plos.org/protocols?utm_medium=editorial-email&utm_source=authorletters&utm_campaign=protocols.

As the corresponding author, your ORCID iD is verified in the submission system and will appear in the published article. PLOS supports the use of ORCID, and we encourage all coauthors to register for an ORCID iD and use it as well. Please encourage your coauthors to verify their ORCID iD within the submission system before final acceptance, as unverified ORCID iDs will not appear in the published article. *Only*  the individual author can complete the verification step; PLOS staff *cannot*  verify ORCID iDs on behalf of authors.

We look forward to receiving your revised manuscript.

Kind regards,

Eleni Magira

Academic Editor

PLOS One

Journal Requirements:

When submitting your revision, we need you to address these additional requirements.

1. Please ensure that your manuscript meets PLOS ONE's style requirements, including those for file naming. The PLOS ONE style templates can be found at

https://journals.plos.org/plosone/s/file?id=wjVg/PLOSOne_formatting_sample_main_body.pdf and

https://journals.plos.org/plosone/s/file?id=ba62/PLOSOne_formatting_sample_title_authors_affiliations.pdf

2. Thank you for stating the following in the Competing Interests/Financial Disclosure section:

Competing interests:

GD received fees for lectures from AOP Pharma.

MB has no disclosure to declare

AT has no disclosure to declare

SR has no disclosure to declare

NB has no disclosure to declare

JFT received grants from Merck and Pfizer for research unit, consulting fees from Advanz, Pfizer, Menarini and Merck serves as lecturer for Mundipharma and Biomerieux.

ML serves as lecturer for Grifols, Shinogi and consultant for AOP Pharma, Viatris, Previa.

We note that one or more of the authors are employed by commercial companies: AOP Pharma, Advanz, Pfizer, Menarini, Merck, Mundipharma, Biomerieux, Grifols, Shinogi, Viatris, and Previa

a. Please provide an amended Funding Statement declaring this commercial affiliation, as well as a statement regarding the Role of Funders in your study. If the funding organization did not play a role in the study design, data collection and analysis, decision to publish, or preparation of the manuscript and only provided financial support in the form of authors' salaries and/or research materials, please review your statements relating to the author contributions, and ensure you have specifically and accurately indicated the role(s) that these authors had in your study. You can update author roles in the Author Contributions section of the online submission form.

Please also include the following statement within your amended Funding Statement.

“The funder provided support in the form of salaries for authors [insert relevant initials], but did not have any additional role in the study design, data collection and analysis, decision to publish, or preparation of the manuscript. The specific roles of these authors are articulated in the ‘author contributions’ section.”

If your commercial affiliation did play a role in your study, please state and explain this role within your updated Funding Statement.

b. Please also provide an updated Competing Interests Statement declaring this commercial affiliation along with any other relevant declarations relating to employment, consultancy, patents, products in development, or marketed products, etc.

Within your Competing Interests Statement, please confirm that this commercial affiliation does not alter your adherence to all PLOS ONE policies on sharing data and materials by including the following statement: "This does not alter our adherence to PLOS ONE policies on sharing data and materials.” (as detailed online in our guide for authors http://journals.plos.org/plosone/s/competing-interests) . If this adherence statement is not accurate and there are restrictions on sharing of data and/or materials, please state these. Please note that we cannot proceed with consideration of your article until this information has been declared.

Please include both an updated Funding Statement and Competing Interests Statement in your cover letter. We will change the online submission form on your behalf.

3. One of the noted authors is a group or consortium [EUROBACT - 2 Study Group]. In addition to naming the author group, please list the individual authors and affiliations within this group in the acknowledgments section of your manuscript. Please also indicate clearly a lead author for this group along with a contact email address.

4. We note that you have indicated that there are restrictions to data sharing for this study. For studies involving human research participant data or other sensitive data, we encourage authors to share de-identified or anonymized data. However, when data cannot be publicly shared for ethical reasons, we allow authors to make their data sets available upon request. For information on unacceptable data access restrictions, please see http://journals.plos.org/plosone/s/data-availability#loc-unacceptable-data-access-restrictions.

Before we proceed with your manuscript, please address the following prompts:

a) If there are ethical or legal restrictions on sharing a de-identified data set, please explain them in detail (e.g., data contain potentially identifying or sensitive patient information, data are owned by a third-party organization, etc.) and who has imposed them (e.g., a Research Ethics Committee or Institutional Review Board, etc.). Please also provide contact information for a data access committee, ethics committee, or other institutional body to which data requests may be sent.

b) If there are no restrictions, please upload the minimal anonymized data set necessary to replicate your study findings to a stable, public repository and provide us with the relevant URLs, DOIs, or accession numbers. Please see http://www.bmj.com/content/340/bmj.c181.long for guidelines on how to de-identify and prepare clinical data for publication. For a list of recommended repositories, please see https://journals.plos.org/plosone/s/recommended-repositories. You also have the option of uploading the data as Supporting Information files, but we would recommend depositing data directly to a data repository if possible.

Please update your Data Availability statement in the submission form accordingly.

5. Please ensure that you refer to Figure 1 in your text as, if accepted, production will need this reference to link the reader to the figure.

6. We note you have included a table to which you do not refer in the text of your manuscript. Please ensure that you refer to Table 3 in your text; if accepted, production will need this reference to link the reader to the Table.

7. Please include your tables as part of your main manuscript and remove the individual files. Please note that supplementary tables (should remain/ be uploaded) as separate "supporting information" files

8. Please include captions for your Supporting Information files at the end of your manuscript, and update any in-text citations to match accordingly. Please see our Supporting Information guidelines for more information: http://journals.plos.org/plosone/s/supporting-information.

9. If the reviewer comments include a recommendation to cite specific previously published works, please review and evaluate these publications to determine whether they are relevant and should be cited. There is no requirement to cite these works unless the editor has indicated otherwise.

Additional Editor Comments:

This study uses data from the EUROBACT-2 cohort to evaluate the effect of hydrocortisone on day-28 mortality in ICU patients with hospital-acquired bloodstream infections (HA-BSI). Its a large, multicenter international database, that appropriately distinguishes between sepsis and septic shock using Sepsis-3 definitions, and incorporates validated severity scoring systems such as SAPS II and SOFA. The application of directed acyclic graphs and propensity score matching helps reduce confounding by indication. Several important points are needed attention. Important variables influencing the decision to administer hydrocortisone—such as clinician judgment, hemodynamic instability beyond recorded norepinephrine dose, or timing of shock onset—are not be fully captured. Please provided these data. Also, in the matched cohort, hydrocortisone use was associated with increased day-28 mortality (51% vs. 41%; OR 1.44, borderline significance -fragile result ) but persisted only in the female subgroup and disappeared after excluding SARS-CoV-2 patients, suggesting not robust causal relationship. Higher mortality in the hydrocortisone group despite matching might strongly suggests that hydrocortisone may be a marker of severity, not a cause of harm. Hydrocortisone exposure is poorly characterized like no precise timing of initiation relative to sepsis onset, no dose variability (most assumed 200 mg, but not confirmed per patient), duration varies (shock resolution vs fixed duration) which is possible to create further heterogeneous exposure groups and the perception that patients must survive to receive treatment. Also, duration beyond total days, and use of adjunctive therapies (e.g., fludrocortisone) introduces heterogeneity in the exposure. This makes it difficult to interpret the true treatment effect and may dilute or bias results. Practice variability influencing treatment decisions as certain centers don’t use hydrocortisone. Please provide a clear justification for your matching strategy, including details on balance diagnostics and the robustness of your findings to alternative analytical approaches.

In general, this is a well-conducted observational study, but the conclusions should be interpreted with caution as hydrocortisone does not clearly improve outcomes in HA-BSI and no strong evidence of harm in septic shock patients

[Note: HTML markup is below. Please do not edit.]

Reviewers' comments:

Reviewer's Responses to Questions

**Comments to the Author**

1. Is the manuscript technically sound, and do the data support the conclusions?

Reviewer #1: Yes

Reviewer #2: Yes

Reviewer #3: Partly

**********

2. Has the statistical analysis been performed appropriately and rigorously?

Reviewer #1: Yes

Reviewer #2: Yes

Reviewer #3: No

**********

3. Have the authors made all data underlying the findings in their manuscript fully available?

The PLOS Data policy

Reviewer #1: Yes

Reviewer #2: Yes

Reviewer #3: Yes

**********

4. Is the manuscript presented in an intelligible fashion and written in standard English?

Reviewer #1: Yes

Reviewer #2: Yes

Reviewer #3: Yes

**********

Reviewer #1: The authors used the database of the EUROBACT-2 cohort to investigate the effects of hydrocortisone in sepsis patients with hospital-acquired bloodstream infections. The database included data from 2600 patients and 333 intensive care units in 52 countries. A 1:1 propensity score was used to match septic patients with and without hydrocortisone, followed by a multivariate analysis with logistical regression. The authors found that the use of hydrocortisone was associated with increased 28-day mortality in patients with nosocomial bacteremia. After excluding patients with SARS-CoV-2 infection, this association could not be confirmed, except for the female subgroup, in whom increased mortality was observed with hydrocortisone.

Comments

The world-wide multi-national cohort EUROBACT-2 study was an observational study on patients with hospital-acquired blood-stream infection and sepsis. This patient group with nosocomial infection has a significantly higher mortality risk (approximately twice as high) than patients in typical sepsis cohorts with mixed, primarily community-acquired underlying infections. Therefore, the group studied represents a high-risk group that differs substantially from patients in randomized controlled trials (RCTs) investigating the treatment of septic shock with steroids. This important information should be mentioned in the introduction.

A known problem with world-wide multinational studies on defined clinical entity is that different health systems and treatment methods in individual countries may lead to different results. This phenomenon became evident in the ADRENAL study, a double-blind study which investigated treatment of hydrocortisone in septic shock: 28-day mortality was significantly lower in Australia on its own, but this difference no longer existed when combined with the results from other countries (NEJM 2018, Figure S5: Mortality by region). Despite a standardized intervention and uniform inclusion criteria, the results differed from country to country.

The EUROBACT-2 study faces the same problem. It has been shown that center and country factors impact on outcome in the EUROBACT-2 cohort (Buetti N, Tabah A, et al. The role of centre and country factors on process and outcome indicators in critically ill patients with hospital-acquired bloodstream infections. Intensive Care Med. 2024;50(6):873-89). Mortality and the decision to forgo life-sustaining treatment were influenced by the country's health expenditure. Centers with better staffing ratios showed improved process indicators and outcome. For this reason, using the EUROBACT-2 dataset to investigate interventions based on outcome endpoints can lead to significant selection bias mainly based on bias in clinical practices in different centers and countries.

The authors attempt to compensate for this problem through 1:1 propensity score matching mimicking an RCT as proposed by Peter C. Austin (2011). The authors write that matching was achieved using nearest neighbor matching with a 1:1 ratio without replacement and with a caliper width of 0.25 SD of the propensity score. Observed baseline characteristics like gender, Charlson comorbidity score, diagnosis at ICU admission, SOFA score (without cardiovascular component) on ICU admission, septic shock, and country of included center were used for propensity matching. However, as good the propensity score matching method may be, it is questionable whether country and center effects are adequately compensated for. Since the database contained data from 2,600 patients and 333 intensive care units in 52 countries, it is of interest to the reader to know whether the nearest neighbor, after matching, comes from the same center or at least from the same country. Only in this way could the reported differences in healthcare systems be reconciled.

As can be seen from the tables in the supplement, there is evidence that patients treated with hydrocortisone were more severely ill. Even after propensity score matching, differences are observed in SOFA score, maximum lactate levels, the use of invasive ventilation and ECMO. Not all differences were adequately compensated for after propensity score matching, as shown by differences in the maximum lactate, a valid marker for the mortality risk of sepsis. For this reason, it seems problematic to form two identical groups from the existing data set that can be compared with regard to their outcome.

Despite this significant limitation, the information in this analysis is valuable and should be reported. Harmful effects of hydrocortisone in septic shock are possible and must always be considered. However, it is important to place this analysis in the correct context. Here are some suggestions for revision:

1) Despite their clinical relevance, several critical and previously reported limitations of the EUROBACT-2 study should be mentioned in the introduction. It should be noted that the study was conducted during the COVID pandemic and that profound economic and regional differences make a general interpretation of the results difficult, and how the authors attempt to take this into account in their analysis.

2) It is well demonstrated that patients with hospital-acquired sepsis face a much higher risk of mortality (approximately twice the risk of death) than those who develop sepsis in the community. In this analysis, the mean time between initial hospital admission and diagnosis of hospital-acquired bloodstream infection was 18 ± 29 days. Therefore, the studied group represents a high-risk group that differs from published studies testing the treatment of septic shock with steroids with a high rate of community-acquired infections. This should be highlighted in the introduction and discussed as a limitation on the generalizability of this analysis.

3) Results: The authors report that administration of hydrocortisone is inconsistent; some centers do not use it at all. Hydrocortisone was administered in 73% of cases until the shock subsided, but only 17.5% were treated for 7 days. This differs significantly from the intervention in RCTs, which may have led to considerably shorter intervention times. This could have influenced the results obtained. How did the authors attempt to address this problem of unproved intervention?

4) Results: Centers using fludrocortisone with hydrocortisone showed a trend to the improvement of survival. Further details (country, duration of steroid treatment) about this subgroup are of interest.

5) Results: Norepinephrine equivalent dose of 0.25µg/kg/min or more showed increased mortality which is against the current study results and recommendation. This strongly suggests that high doses of noradrenaline were also used as a criterion for limiting therapy in cases of protracted critical illness.

6) Discussion: The ADRENAL trial reports gender differences regarding secondary endpoints (Thompson K, et al. on behalf of the Adrenal Investigators s-daSC. Sex differences in response to adjunctive corticosteroid treatment for patients with septic shock. Intensive Care Med. 2021;47(2):246-8.). Women are more likely to experience recurrent shock while receiving hydrocortisone, whereas men had significantly shorter times to discharge from the intensive care unit and to weaning from mechanical ventilation. This context should be discussed.

7) Discussion: Since this analysis focuses on the intervention with hydrocortisone, similar studies with dexamethasone (different steroid, no mineralocorticoid effect) should not be included into the discussion.

8) Discussion: The authors did not discuss the distinction between early-onset sepsis (EOS) and late-onset sepsis (LOS) which involves a fundamental shift in the host’s immunological state. The cohort under study is a typical LOS cohort. The most defining feature of LOS - particularly nosocomial bloodstream infections - is immunoparalysis characterized by low monocyte human leukocyte antigen-DR expression and endotoxin tolerance. It is quite conceivable that steroids could cause undesirable side effects in patients with immunoparalysis.

Reviewer #2: General comments

The authors assessed the impact of hydrocortisone use on outcomes in critically ill patients with documented healthcare-associated bloodstream infections (HA-BSI), using data from the EUROBACT-2 cohort. Patients who did and did not receive hydrocortisone were matched using a propensity score approach. In the multivariable analysis of the matched cohort, hydrocortisone use was associated with an increased risk of day-28 mortality. This association was also observed among females in the overall cohort. In a sensitivity analysis excluding patients with COVID-19, hydrocortisone was not associated with day-28 mortality overall, but remained associated with increased mortality in females.

The manuscript is well written and easy to follow. However, I have several concerns regarding the methodology and the interpretation of the results. In particular, it would be helpful if the authors could clarify how key relationships were considered in the construction of the DAG and discuss the potential implications for variable selection and model adjustment.

Major comments

The DAG appropriately represents source control and timing of adequate antimicrobial therapy as distinct variables. However, the relationships between these variables, treatment allocation, and disease severity are not fully specified.

- 1. Source control is an important determinant of outcomes in patients with septic shock, although its incorporation into the analytical framework can be challenging. In clinical practice, source control may not always be feasible in the most unstable patients, for example due to constraints related to transport or surgical intervention. Conversely, initial treatments such as hydrocortisone and antibiotics may lead to transient clinical improvement, potentially enabling source control that was not initially possible. In this context, the role of source control within the model may be complex. It would be helpful if the authors could provide additional clarification on how this variable was handled and discuss its potential implications for the interpretation of the findings.

- 2. While septic shock is included in the model, the absence of an explicit link between norepinephrine dose and hydrocortisone initiation may still be a limitation of the current causal framework. In clinical practice, vasopressor requirements—particularly at commonly used thresholds such as 0.25 µg/kg/min—often influence the decision to initiate hydrocortisone, beyond the binary classification of septic shock. It would therefore be helpful if the authors could clarify how vasopressor dose was considered in the construction of the DAG and discuss the potential implications for variable selection and adjustment.

- 3. The authors included the number of days of hydrocortisone administration as a confounding factor in the model. However, as this variable is likely to occur after treatment initiation, it may reflect, at least in part, a post-exposure factor rather than a baseline confounder. In this context, adjusting for it could potentially influence the estimates. It would therefore be helpful if the authors could clarify how this variable was considered in the analysis and more clearly distinguish baseline confounders from post-exposure variables.

- 4. While propensity score matching improves comparability by excluding patients with non-overlapping scores, it also restricts the analysis to a subset of patients. It may therefore be helpful to provide the characteristics of excluded patients and discuss the potential implications for the generalizability of the findings.

5. Clarification would be appreciated regarding whether the caliper was applied to the raw propensity score or to the logit of the propensity score, as the latter is more commonly recommended.

Minor comments

- 1. The authors state in the Methods section that “a cut-off of >0.25 µg/kg/min was used to define high-dose norepinephrine, as recommended by SSC guidelines [1].” However, the cited SSC passage appears to correspond to a practice remark rather than a formal definition or recommendation. Clarification of this point would be helpful.

- 2. Regarding the variable “time to adequate antibiotic therapy,” the use of “never” as the reference category may reflect an attempt to define a more homogeneous comparator group, particularly if the dataset did not allow a sufficiently precise assessment of early treatment delays. Nevertheless, this choice may limit the clinical interpretability of the analysis, especially in patients with septic shock, in whom even short delays in appropriate antimicrobial therapy may influence outcomes. It would therefore be helpful if the authors could clarify the rationale for this choice and further discuss its potential implications in the Discussion section.

- 3. An additional sensitivity analysis excluding coagulase-negative staphylococci might have been informative. Although these organisms may represent true bloodstream infections in some patients, their clinical significance can be more heterogeneous than that of other pathogens. Excluding them in a sensitivity analysis could therefore help assess whether the main findings remain consistent in a population with less microbiological ambiguity.

Overall, this is an interesting and clinically relevant study. Addressing some of the points raised above may help to further clarify the methodology and support the interpretation of the findings.

Reviewer #3: Thank you for giving me the opportunity to review the article named « Effect of hydrocortisone on the outcome of sepsis patients with hospital-acquired bloodstream infections: an ancillary case - control study of EUROBACT-2 cohort ».

This study aims to evaluate the effect of hydrocortisone on day-28 mortality in septic patients with HA-BSI, a population in which corticosteroids have not been specifically investigated despite current recommendations in septic shock.

Using data from the prospective EUROBACT-2 cohort, the authors performed a 1:1 propensity score matching between patients receiving hydrocortisone and controls, followed by multivariate logistic regression. Finally in the whole cohort, hydrocortisone use was associated with an increased risk of day-28 mortality. This association was particularly observed in females. This is a relevant clinical question, particularly given the ongoing uncertainty regarding corticosteroid use outside septic shock.

The use of the EUROBACT-2 cohort is a strength, especially given the microbiological documentation of infection. However, several important methodological issues and clarifications to improve the manuscript.

General comments

Introduction

The rationale of the study needs to be strengthened. The authors should better justify:

• why hydrocortisone is assessed in nosocomial sepsis (HA-BSI) rather than specifically in septic shock, where its use is currently recommended,

• and more broadly, why evaluating corticosteroids in sepsis without shock is clinically relevant.

In addition, the manuscript should clarify why focusing on hospital-acquired bloodstream infections would provide a specific advantage compared to other infectious contexts.

Methods

The inclusion criteria are currently unclear and need to be carefully revised. As written, it suggests that only patients receiving hydrocortisone were included, which is inconsistent with the study objective comparing exposed and non-exposed patients.

The definition of exposure to hydrocortisone also requires clarification:

• What was the timing between hydrocortisone initiation and bloodstream infection diagnosis?

• Was there a predefined time window (e.g., within ±48 hours of blood culture positivity)?

It would also be important to distinguish:

• bloodstream infections present at ICU admission,

• from ICU-acquired HA-BSI.

Additional clarifications are needed regarding:

• the delay between blood culture positivity and ICU admission for patients with ward-acquired infections,

• whether data (severity scores, organ support) were collected at ICU admission or at the time of HA-BSI diagnosis.

The manuscript should also specify:

• the type of corticosteroid corresponding to “20 mg/day” (e.g., prednisone equivalent),

• whether patients receiving corticosteroids other than hydrocortisone (e.g., dexamethasone for pneumonia or COVID-19) were excluded.

Regarding septic shock definition, please confirm whether:

• lactate >2 mmol/L was required (Sepsis-3 definition),

• and provide details on lactate data availability (granularity of the database).

A subgroup analysis based on vasopressor requirement (e.g., norepinephrine >0.25 µg/kg/min) would be of interest, given current guideline recommendations.

Statistical analysis

The statistical approach requires clarification.

• It is unclear whether a double “matching” was achieved: one with PS and the other with mentioned covariates.

• Please clearly describe:

o variables included in the propensity score,

o other variables used for matching?

o variables included in the final model.

• The inclusion of “duration of hydrocortisone administration” as a covariate is questionable, as it is likely influenced by clinical evolution (e.g., duration of shock) and may introduce bias. This variable should be reconsidered.

Please provide:

• a visualization of the propensity score distribution across groups,

• standardized mean differences (SMDs) before and after matching to assess balance.

Please justify the analysis of the female subgroup. Specifically:

• clarify whether this analysis was pre-specified or post hoc;

• if it was pre-specified, provide the underlying rationale and incorporate it into the Introduction;

• if it was post hoc, clearly state this and interpret the findings with appropriate caution.

Results

• The section titled “Characteristics of corticosteroid use according to centre” should be renamed to:

→ “Characteristics of corticosteroid administration”

• Even after matching, patients receiving hydrocortisone appear to have:

o higher vasopressor requirements,

o more organ support.

� This suggests residual confounding and should be explicitly addressed.

• Additional clinically relevant outcomes would strengthen the manuscript, such as:

o vasopressor-free days,

o timing for shock resolution,

o recurrence of nosocomial infection.

• Minor correction:

o Typo error: “In In the final analysis” → “In the final analysis”

Discussion

The discussion should be strengthened regarding : potential confounding by indication,

• time-dependent bias related to corticosteroid exposure,

• residual confounding despite matching,

• and heterogeneity of sepsis phenotypes.

Finally, the interpretation of results should be more cautious. The observed association between hydrocortisone and mortality should not be interpreted as causal given the observational design.

**********

what does this mean?). If published, this will include your full peer review and any attached files.

If you choose “no”, your identity will remain anonymous but your review may still be made public.

**Do you want your identity to be public for this peer review?**  For information about this choice, including consent withdrawal, please see our Privacy Policy

Reviewer #1: **Yes:** Josef Briegel

Reviewer #2: No

Reviewer #3: No

**********

[NOTE: If reviewer comments were submitted as an attachment file, they will be attached to this email and accessible via the submission site. Please log into your account, locate the manuscript record, and check for the action link "View Attachments". If this link does not appear, there are no attachment files.]

To ensure your figures meet our technical requirements, please review our figure guidelines: https://journals.plos.org/plosone/s/figures

You may also use PLOS’s free figure tool, NAAS, to help you prepare publication quality figures: https://journals.plos.org/plosone/s/figures#loc-tools-for-figure-preparation.

NAAS will assess whether your figures meet our technical requirements by comparing each figure against our figure specifications.

# Author comment: Author response to Decision Letter 1

**DOI:** 10.1371/journal.pone.0356721.r002

4 Jun 2026

Detailed Response to Reviewers

EUROBACT/hydrocortisone manuscript — expanded point-by-point response

Dear Academic Editor and Reviewers,

We sincerely thank the Academic Editor and the reviewers for their careful reading of our manuscript and for the constructive comments that helped us improve both the methodological transparency and the interpretation of our work. We have substantially revised the statistical analysis, the presentation of the results, and the wording of the manuscript. In particular, we strengthened the propensity score model, clarified the matching strategy, added balance diagnostics, removed post-exposure hydrocortisone duration from the main model, performed several sensitivity analyses, and toned down causal language throughout the manuscript. Below, we provide a point-by-point response and summarize the concrete actions performed for each issue raised.

Journal requirements

Comment 1. Formatting, figure/table citations, and supporting information.

Response. Thank you for outlining this requirement. We revised the manuscript formatting according to PLOS ONE requirements. Main tables are now intended to be included in the manuscript file; supplementary tables and figures have been prepared as supporting information with explicit captions. We also checked that all figures and tables are cited in the text, including Figure 1 and Table 3. The revised submission includes a clean manuscript, a marked-up manuscript, and a separate response-to-reviewers file.

Comment 2. Funding and competing interests.

Response. We thank the journal office for this clarification. We have provided updated Funding and Competing Interests statements in the cover letter and online submission form. The updated statements clarify that the declared relationships did not influence study design, data collection, data analysis, interpretation, decision to publish, or manuscript preparation. We have also included the required PLOS ONE statement that these declarations do not alter our adherence to PLOS ONE policies on sharing data and materials.

Comment 3. EUROBACT-2 Study Group.

Response. Thank you for this administrative point. We have updated the acknowledgments to list EUROBACT-2 Study Group contributors and affiliations and to identify a lead contact for the group, in accordance with PLOS ONE requirements.

Comment 4. Data availability.

Response. We have addressed this requirement by revising the Data Availability Statement. Because the dataset contains sensitive individual-level clinical data collected across multiple countries and institutions, unrestricted public sharing is not permitted. The revised statement provides the appropriate data-access contact and specify conditions under which qualified researchers may request access.

Comment 5. Ethics statement.

Response. We appreciate this clarification. The previous submission form entry of 'N/A' was inconsistent with the manuscript. The revised submission states that EUROBACT-2 was approved as a low-risk research project with waiver of individual consent by the Human Research Ethics Committee of the Royal Brisbane & Women’s Hospital, Queensland, Australia (LNR/2019/QRBW/48376), with local approvals obtained according to national and local regulations.

Response to the Academic Editor

Comment 1. Important variables influencing hydrocortisone administration may not have been fully captured.

Response. We thank the Academic Editor for this important comment. We strengthened the propensity score model by adding age, lactate at HA-BSI diagnosis, and ICU-acquired versus non-ICU-acquired HA-BSI to the original variables. The final PS included age, sex, Charlson score, ICU admission diagnosis, SOFA without cardiovascular component, septic shock, country, lactate at HA-BSI diagnosis, and ICU-acquired/non-ICU-acquired HA-BSI. Matching used 1:1 nearest-neighbour matching without replacement and a caliper of 0.2 SD of the logit PS, resulting in 521 pairs and 1,042 matched patients. We also added love plots and explicitly acknowledge that clinician judgment, unmeasured hemodynamic instability and timing of shock onset may remain residual confounders.

Comment 2. The main association was borderline and appeared fragile in the original analysis.

Response. We are grateful for this careful assessment. We repeated the analysis using the revised PS and updated multivariable model. In the revised main analysis, hydrocortisone remained associated with day-28 mortality in the overall matched cohort. The association also persisted after excluding COVID-19 patients. Importantly, the association was not observed among patients with septic shock or among those receiving norepinephrine-equivalent dose ≥0.25 µg/kg/min. We revised the interpretation to state that the signal appears mainly outside the subgroup closest to guideline-based hydrocortisone indications.

Comment 3. Hydrocortisone may be a marker of severity rather than a cause of harm.

Response. Thank you for raising this important methodological point. We removed causal wording throughout the manuscript. The results are now described as associations. The Discussion explicitly addresses confounding by indication, residual confounding, and the possibility that hydrocortisone use may reflect perceived severity rather than causing harm.

Comment 4. Hydrocortisone exposure is poorly characterized, especially timing of initiation.

Response. We appreciate this comment, which helped us clarify a key limitation. The exact date or time of hydrocortisone initiation relative to blood culture sampling or HA-BSI diagnosis was not available in EUROBACT-2. We therefore cannot analyze the temporal relationship between exposure and infection diagnosis. This has been added to the Methods and Discussion, together with the statement that time-dependent bias and residual time-dependent confounding cannot be excluded.

Comment 5. Hydrocortisone duration varies and may create heterogeneous exposure groups.

Response. We thank the Academic Editor for highlighting this issue. In the initial version of the manuscript, duration of hydrocortisone exposure was included in the multivariable model. After reconsideration, and in response to the concern raised during the review process, we agree that this variable should not be included in the primary adjustment model, as it occurs after treatment initiation and may be influenced by subsequent clinical evolution and survival. We therefore removed hydrocortisone duration from the primary multivariable model. With this revised and methodologically more appropriate model, hydrocortisone use was associated with day-28 mortality in the overall matched cohort. This finding remains observational and should not be interpreted causally. Hydrocortisone duration is now considered descriptively rather than as a baseline confounder.

Comment 6. Practice variability and centre effects may influence treatment decisions.

Response. This is an important point. Country was included in the PS model, and we added a dedicated love plot for centre/country/income variables. Same-centre or same-country exact matching was not feasible without major sample loss, and this is acknowledged as a limitation. We now explicitly discuss residual centre- and country-level confounding.

Comment 7. Please justify the matching strategy and robustness of findings.

Response. We clarified the matching strategy in the Methods: 1:1 nearest-neighbour matching without replacement, caliper 0.2 SD of the logit PS. We added PS distribution plots and three love plots. We also performed several sensitivity analyses: excluding COVID-19 patients, excluding CoNS patients from the already matched cohort, removing time to adequate antimicrobial therapy and source control from the model, analyzing the high-dose norepinephrine subgroup, and exploring secondary outcomes.

Comment 8. The conclusion should be cautious.

Response. We thank the Academic Editor for this important comment. The conclusion has been revised to state that hydrocortisone use was associated with higher day-28 mortality in the overall matched cohort, but not among patients with septic shock or high-dose norepinephrine. We explicitly describe the findings as observational and hypothesis-generating.

Response to Reviewer 1

Comment 1. The introduction should highlight that HA-BSI is a high-risk population differing from typical sepsis RCT cohorts.

Response. We thank Reviewer 1 for this helpful comment. We revised the Introduction to emphasize that HA-BSI is a high-risk, hospital-acquired and microbiologically documented infection context. We also clarified that this population differs from many randomized trials of corticosteroids in septic shock, which often included broader sepsis populations and variable microbiological documentation.

Comment 2. Worldwide studies are affected by healthcare-system, country and centre effects.

Response. Thank you for this important suggestion. We addressed this by including country in the PS model and by presenting centre/country/income balance in a dedicated love plot. We also expanded the Discussion to acknowledge residual country- and centre-level confounding.

Comment 3. It would be useful to know whether matched pairs came from the same centre or country.

Response. We appreciate the reviewer’s careful reading. We assessed this issue in the final matched cohort. Exact same-centre or same-country matching was not used because it would have produced major sample loss. The revised manuscript clarifies that country was addressed through the PS model and that residual centre/country effects remain possible.

Comment 4. Patients receiving hydrocortisone appeared more severely ill even after matching.

Response. We agree that this needed further clarification. We added lactate at HA-BSI diagnosis and ICU-acquired/non-ICU-acquired HA-BSI to the PS model. We also added love plots separating PS/multivariable variables, centre/country variables, and clinically relevant variables not included in the model, including organ support and severity markers.

Comment 5. COVID pandemic and economic/regional differences should be mentioned.

Response. This is a very relevant comment. We added this context and performed a sensitivity analysis excluding COVID-19 patients. In this analysis, the global association persisted, but no association was found in patients with septic shock or high-dose norepinephrine.

Comment 6. Hydrocortisone administration differed across centres and duration differed from RCTs.

Response. We thank the reviewer for this comment. We now describe real-life treatment heterogeneity more clearly. Hydrocortisone duration was removed from the main model because it is post-exposure and may reflect clinical evolution.

Comment 7. Centres using fludrocortisone require further description, including country and duration.

Response. We performed a descriptive analysis of fludrocortisone use. It was limited to a small number of centres and countries, mostly France, and involved relatively few hydrocortisone-treated patients. Crude mortality was numerically lower in fludrocortisone-using centres but not statistically significant, and hydrocortisone duration was broadly similar. We use this only to acknowledge treatment heterogeneity and do not draw conclusions on fludrocortisone efficacy.

Comment 8. High-dose norepinephrine may reflect severity or limitation of therapy.

Response. We performed a dedicated subgroup analysis among patients receiving norepinephrine-equivalent dose ≥0.25 µg/kg/min. Hydrocortisone was not associated with day-28 mortality in this subgroup. This is now highlighted because this subgroup is closest to current guideline-based corticosteroid indications.

Comment 9. Discuss ADRENAL sex-related findings.

Response. We added a cautious discussion of sex-related findings. However, the formal hydrocortisone × sex interaction test was not significant, so the female subgroup signal is presented as exploratory.

Comment 10. Avoid dexamethasone studies when discussing hydrocortisone.

Response. We revised the Discussion to focus on hydrocortisone and hydrocortisone/fludrocortisone evidence and avoided overinterpreting dexamethasone studies.

Comment 11. Discuss late-onset sepsis and immunoparalysis.

Response. We added a cautious discussion of late-onset/hospital-acquired sepsis and possible immune dysfunction in prolonged critical illness, without presenting this as a mechanism demonstrated in our data.

Response to Reviewer 2

Comment 1. Clarify source control handling, given its complex relation with severity and outcomes.

Response. We thank Reviewer 2 for this important methodological comment. Source control was retained in the main model because it is prognostically important, but we now discuss its potentially post-baseline nature. We also performed a sensitivity analysis removing both source control and time to adequate antimicrobial therapy; the overall hydrocortisone association persisted, suggesting that the main result was not solely driven by these covariates.

Comment 2. Clarify norepinephrine dose in the causal framework and model.

Response. Thank you for this thoughtful remark. Norepinephrine-equivalent dose ≥0.25 µg/kg/min was included as a multivariable covariate, and a dedicated subgroup analysis was performed. Hydrocortisone was not associated with mortality in this high-dose norepinephrine subgroup.

Comment 3. Hydrocortisone duration is post-exposure and should be reconsidered.

Response. We thank the reviewer for this important methodological comment. In the initial version of the manuscript, duration of hydrocortisone exposure was included in the multivariable model. After reconsideration, and in response to the reviewer’s concern, we agree that this variable should not be included in the primary adjustment model, as it occurs after treatment initiation and may be influenced by subsequent clinical evolution and survival. We therefore removed hydrocortisone duration from the primary multivariable model. With this revised and methodologically more appropriate model, hydrocortisone use was associated with day-28 mortality in the overall matched cohort. This result remains observational and should not be interpreted causally.

Comment 4. Provide characteristics of patients excluded by matching and discuss generalizability.

Response. We added a supplementary matched-versus-unmatched analysis among patients complete for the final PS. This includes matched vs unmatched patients overall, matched vs unmatched hydrocortisone-treated patients, and matched vs unmatched controls. Both absolute SMDs and p-values are provided. This analysis shows that the matched results apply to the common-support population, not necessarily the entire EUROBACT-2 HA-BSI cohort.

Comment 5. Clarify whether the caliper was applied to raw PS or logit PS.

Response. We clarified that matching used a caliper of 0.2 SD of the logit propensity score.

Comment 6. The SSC norepinephrine threshold should be described as a practice remark rather than a formal definition.

Response. We revised the wording accordingly and now describe the norepinephrine-equivalent threshold as a clinically relevant guideline-based practice threshold rather than a formal definition.

Comment 7. The 'never' reference category for adequate antimicrobial therapy may limit interpretability.

Response. We clarified the categorization and reference category. We also performed a sensitivity analysis excluding time to adequate antimicrobial therapy together with source control; the global hydrocortisone association persisted.

Comment 8. Perform sensitivity analysis excluding coagulase-negative staphylococci.

Response. We performed this sensitivity analysis from the already matched cohort, without rematching. After excluding patients with CoNS infection, the global hydrocortisone association persisted, whereas no association was observed among patients with septic shock or high-dose norepinephrine. This suggests that the findings are not solely explained by inclusion of CoNS infections.

Response to Reviewer 3

Comment 1. Strengthen rationale for hydrocortisone in nosocomial sepsis and sepsis without shock.

Response. We first would like to thank reviewer 3 for the time employed to review this work. We revised the Introduction to explai

Attachment Submitted filename: Response_to_Reviewers_EUROBACT_hydrocortisone.docx

# Peer review: Decision Letter 1

**DOI:** 10.1371/journal.pone.0356721.r003

**Authors:** Iddya Karunasagar

26 Jun 2026

Dear Dr. Duclos,

Thank you for submitting your manuscript to PLOS ONE. After careful consideration, we feel that it has merit but does not fully meet PLOS ONE’s publication criteria as it currently stands. Therefore, we invite you to submit a revised version of the manuscript that addresses the points raised during the review process.

==============================

Please make the Figure labelling consistent as pointed out by the reviewer.

==============================

Please submit your revised manuscript by Aug 10 2026 11:59PM. If you will need more time than this to complete your revisions, please reply to this message or contact the journal office at plosone@plos.org. When you're ready to submit your revision, log on to https://www.editorialmanager.com/pone/ and select the 'Submissions Needing Revision' folder to locate your manuscript file.

- A letter that responds to each point raised by the academic editor and reviewer(s). You should upload this letter as a separate file labeled 'Response to Reviewers'.
- A marked-up copy of your manuscript that highlights changes made to the original version. You should upload this as a separate file labeled 'Revised Manuscript with Track Changes'.
- An unmarked version of your revised paper without tracked changes. You should upload this as a separate file labeled 'Manuscript'.

If you would like to make changes to your financial disclosure, please include your updated statement in your cover letter. Guidelines for resubmitting your figure files are available below the reviewer comments at the end of this letter.

If applicable, we recommend that you deposit your laboratory protocols in protocols.io to enhance the reproducibility of your results. Protocols.io assigns your protocol its own identifier (DOI) so that it can be cited independently in the future. For instructions see: https://journals.plos.org/plosone/s/submission-guidelines#loc-laboratory-protocols. Additionally, PLOS ONE offers an option for publishing peer-reviewed Lab Protocol articles, which describe protocols hosted on protocols.io. Read more information on sharing protocols at https://plos.org/protocols?utm_medium=editorial-email&utm_source=authorletters&utm_campaign=protocols.

As the corresponding author, your ORCID iD is verified in the submission system and will appear in the published article. PLOS supports the use of ORCID, and we encourage all coauthors to register for an ORCID iD and use it as well. Please encourage your coauthors to verify their ORCID iD within the submission system before final acceptance, as unverified ORCID iDs will not appear in the published article. *Only*  the individual author can complete the verification step; PLOS staff *cannot*  verify ORCID iDs on behalf of authors.

We look forward to receiving your revised manuscript.

Kind regards,

Iddya Karunasagar

Academic Editor

PLOS One

Journal Requirements:

1. If the reviewer comments include a recommendation to cite specific previously published works, please review and evaluate these publications to determine whether they are relevant and should be cited. There is no requirement to cite these works unless the editor has indicated otherwise.

2. Please review your reference list to ensure that it is complete and correct. If you have cited papers that have been retracted, please include the rationale for doing so in the manuscript text, or remove these references and replace them with relevant current references. Any changes to the reference list should be mentioned in the rebuttal letter that accompanies your revised manuscript. If you need to cite a retracted article, indicate the article’s retracted status in the References list and also include a citation and full reference for the retraction notice.

**Additional Editor Comments:**

Please see the comment of the reviewer regarding labeling variables in some figures for consistency.

[Note: HTML markup is below. Please do not edit.]

Reviewers' comments:

Reviewer's Responses to Questions

**Comments to the Author**

Reviewer #2: All comments have been addressed

Reviewer #3: All comments have been addressed

**********

2. Is the manuscript technically sound, and do the data support the conclusions?

Reviewer #2: Yes

Reviewer #3: Yes

**********

3. Has the statistical analysis been performed appropriately and rigorously?

Reviewer #2: Yes

Reviewer #3: Yes

**********

4. Have the authors made all data underlying the findings in their manuscript fully available?

The PLOS Data policy

Reviewer #2: Yes

Reviewer #3: Yes

**********

5. Is the manuscript presented in an intelligible fashion and written in standard English?

Reviewer #2: Yes

Reviewer #3: Yes

**********

Reviewer #2: Minor comment: In Figures 3 and 5, the names of the variables “time to adequate antibiotic treatment” and “source control” are not displayed, whereas only their categories (e.g., 0–24 h, 24–48 h, controlled/not controlled) are shown. For consistency with Figure 2 and to improve readability, the authors may wish to include the corresponding variable names in these figures.

Reviewer #3: I have no further comments. The authors should be commended for the substantial improvements made in this revised version of the manuscript.

**********

what does this mean?). If published, this will include your full peer review and any attached files.

If you choose “no”, your identity will remain anonymous but your review may still be made public.

**Do you want your identity to be public for this peer review?**  For information about this choice, including consent withdrawal, please see our Privacy Policy

Reviewer #2: **Yes:** bertrand Souweine

Reviewer #3: No

**********

[NOTE: If reviewer comments were submitted as an attachment file, they will be attached to this email and accessible via the submission site. Please log into your account, locate the manuscript record, and check for the action link "View Attachments". If this link does not appear, there are no attachment files.]

To ensure your figures meet our technical requirements, please review our figure guidelines: https://journals.plos.org/plosone/s/figures

You may also use PLOS’s free figure tool, NAAS, to help you prepare publication quality figures: https://journals.plos.org/plosone/s/figures#loc-tools-for-figure-preparation.

NAAS will assess whether your figures meet our technical requirements by comparing each figure against our figure specifications.

# Author comment: Author response to Decision Letter 2

**DOI:** 10.1371/journal.pone.0356721.r004

15 Jul 2026

Dear Reviewers,

Thank you for the time you have spent on this manuscript. Your reviews were useful to improve its quality.

To reviewer 2:

The names of the variables have been corrected in the figure 3 and 5. Thank you

Attachment Submitted filename: Response_to_Reviewers_EUROBACT_hydrocortisone_R2.docx

# Editor report: Decision Letter 2

**DOI:** 10.1371/journal.pone.0356721.r005

**Authors:** Iddya Karunasagar

6 Aug 2026

Association between hydrocortisone use and outcomes in sepsis patients with hospital-acquired bloodstream infections: an ancillary analysis of the EUROBACT-2 cohort

PONE-D-26-00590R2

Dear Dr. Duclos,

We’re pleased to inform you that your manuscript has been judged scientifically suitable for publication and will be formally accepted for publication once it meets all outstanding technical requirements.

Within one week, you’ll receive an e-mail detailing the required amendments. When these have been addressed, you’ll receive a formal acceptance letter and your manuscript will be scheduled for publication.

An invoice will be generated when your article is formally accepted. Please note, if your institution has a publishing partnership with PLOS and your article meets the relevant criteria, all or part of your publication costs will be covered. Please make sure your user information is up-to-date by logging into Editorial Manager at Editorial Manager® and clicking the ‘Update My Information' link at the top of the page. For questions related to billing, please contact billing support.

If your institution or institutions have a press office, please notify them about your upcoming paper to help maximize its impact. If they’ll be preparing press materials, please inform our press team as soon as possible -- no later than 48 hours after receiving the formal acceptance. Your manuscript will remain under strict press embargo until 2 pm Eastern Time on the date of publication. For more information, please contact onepress@plos.org.

Kind regards,

Iddya Karunasagar

Academic Editor

PLOS One

Additional Editor Comments (optional):

All comments have been addressed.

Reviewers' comments:

# Editor report: Acceptance letter

**DOI:** 10.1371/journal.pone.0356721.r006

**Authors:** Iddya Karunasagar

PONE-D-26-00590R2

PLOS One

Dear Dr. Duclos,

I'm pleased to inform you that your manuscript has been deemed suitable for publication in PLOS One. Congratulations! Your manuscript is now being handed over to our production team.

At this stage, our production department will prepare your paper for publication. This includes ensuring the following:

* All references, tables, and figures are properly cited

* All relevant supporting information is included in the manuscript submission,

* There are no issues that prevent the paper from being properly typeset

You will receive further instructions from the production team, including instructions on how to review your proof when it is ready. Please keep in mind that we are working through a large volume of accepted articles, so please give us a few days to review your paper and let you know the next and final steps.

Lastly, if your institution or institutions have a press office, please let them know about your upcoming paper now to help maximize its impact. If they'll be preparing press materials, please inform our press team within the next 48 hours. Your manuscript will remain under strict press embargo until 2 pm Eastern Time on the date of publication. For more information, please contact onepress@plos.org.

You will receive an invoice from PLOS for your publication fee after your manuscript has reached the completed accept phase. If you receive an email requesting payment before acceptance or for any other service, this may be a phishing scheme. Learn how to identify phishing emails and protect your accounts at https://explore.plos.org/phishing.

If we can help with anything else, please email us at customercare@plos.org.

Thank you for submitting your work to PLOS One and supporting open access.

Kind regards,

PLOS ONE Editorial Office Staff

on behalf of

Dr. Iddya Karunasagar

Academic Editor

PLOS One
